# Computer-Driven Development of an in Silico Tool for Finding Selective Histone Deacetylase 1 Inhibitors

**DOI:** 10.3390/molecules25081952

**Published:** 2020-04-22

**Authors:** Hajar Sirous, Giuseppe Campiani, Simone Brogi, Vincenzo Calderone, Giulia Chemi

**Affiliations:** 1 Bioinformatics Research Center, School of Pharmacy and Pharmaceutical Sciences, Isfahan University of Medical Sciences, Isfahan 81746-73461, Iran; 2 Department of Biotechnology, Chemistry and Pharmacy, DoE Department of Excellence 2018–2022, University of Siena, via Aldo Moro 2, 53100 Siena, Italy; campiani@unisi.it (G.C.); GChemi001@dundee.ac.uk (G.C.); 3 Department of Pharmacy, University of Pisa, via Bonanno 6, 56126 Pisa, Italy; vincenzo.calderone@unipi.it

**Keywords:** 3D-QSAR, pharmacophore modeling, ligand-based model, HDACs, isoform-selective histone deacetylase inhibitors, aminophenylbenzamide

## Abstract

Histone deacetylases (HDACs) are a class of epigenetic modulators overexpressed in numerous types of cancers. Consequently, HDAC inhibitors (HDACIs) have emerged as promising antineoplastic agents. Unfortunately, the most developed HDACIs suffer from poor selectivity towards a specific isoform, limiting their clinical applicability. Among the isoforms, HDAC1 represents a crucial target for designing selective HDACIs, being aberrantly expressed in several malignancies. Accordingly, the development of a predictive in silico tool employing a large set of HDACIs (aminophenylbenzamide derivatives) is herein presented for the first time. Software Phase was used to derive a 3D-QSAR model, employing as alignment rule a common-features pharmacophore built on 20 highly active/selective HDAC1 inhibitors. The 3D-QSAR model was generated using 370 benzamide-based HDACIs, which yielded an excellent correlation coefficient value (R^2^ = 0.958) and a satisfactory predictive power (Q^2^ = 0.822; Q^2^_F3_ = 0.894). The model was validated (r^2^_ext_ts_ = 0.794) using an external test set (113 compounds not used for generating the model), and by employing a decoys set and the receiver-operating characteristic (ROC) curve analysis, evaluating the Güner–Henry score (GH) and the enrichment factor (EF). The results confirmed a satisfactory predictive power of the 3D-QSAR model. This latter represents a useful filtering tool for screening large chemical databases, finding novel derivatives with improved HDAC1 inhibitory activity.

## 1. Introduction

Epigenetic defects in gene expression are well known in the onset and progression of cancer. In this context, pharmacological targeting of proteins of the cellular epigenetic machinery has provided opportunities for anti-cancer drug design [1,2]. Among epigenetic enzymes, histone deacetylases (HDACs) hold a fundamental role in regulating gene expression through histone post-translational modifications [3,4,5].

HDACs catalyze the removal of acetyl groups from the acetylated ε-amino termini of lysine residues located at the tails of the nucleosomal histones core. Histone deacetylation process leads to condensed chromatin structure which concomitantly restricts the accessibility of related transcriptional factors to their target genes, thereby suppressing gene expression including tumor suppressor genes [6,7,8]. The abnormal regulation of this process culminates with the high expression level of HDACs. This event has been observed in the development of several human cancers. Consequently, effective inhibition of HDACs has recently gained importance as a valid therapeutic strategy to reverse aberrant epigenetic changes associated with cancer [9,10,11]. HDAC inhibitors (HDACIs) induce histone hyperacetylation and subsequent transcriptional re-activation of suppressed genes which are correlated with a variety of effects on tumor cells including apoptosis, differentiation, cell cycle arrest, inhibition of proliferation and cytostasis [12,13]. 

The HDAC family comprises 18 isoforms in mammalian cells which are categorized into four main classes (class I-IV) based on their structural and functional characteristics. HDACs belonging to class I (HDAC1–3 and 8), II (HDACs 4–7, 9 and 10) and IV (HDAC11) are all zinc-dependent metalloenzymes, while class III HDACs, also known as the sirtuins (SIRT1-7), requires NAD^+^ as a cofactor for their catalytic function [6,14]. 

**Figure 1:**
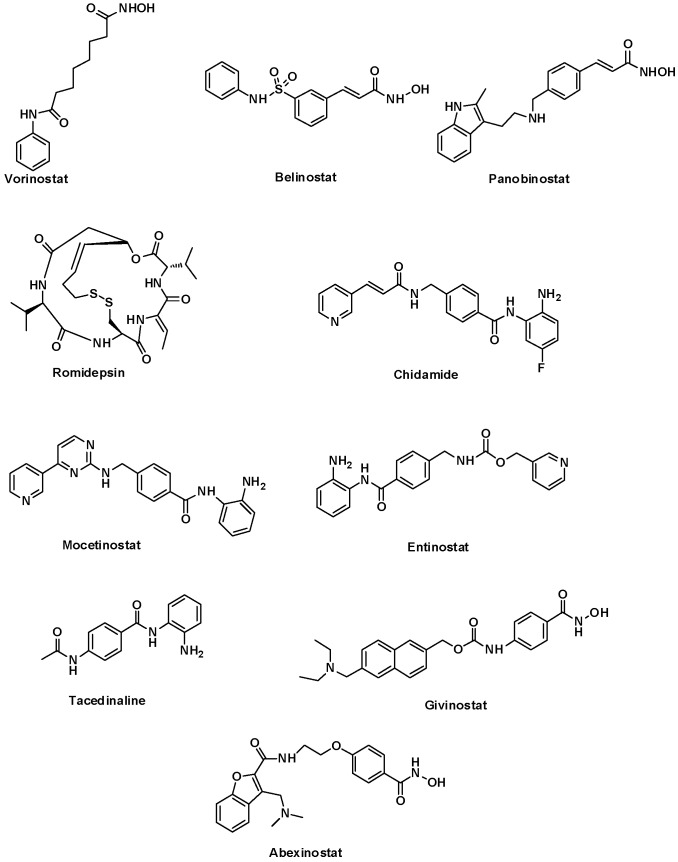
HDACIs approved by FDA and/or in clinical trials.

Extensive efforts over recent decades have led to the identification of four chemically diverse classes of HDACIs as potent antineoplastic agents including, hydroxamates, benzamides, cyclic peptides, and short-chain fatty acids [3]. The main breakthrough in developing these inhibitors was achieved by the US FDA approval of Vorinostat [15], Belinostat [16], Panobinostat (hydroxamate-based inhibitors) [17], Romidepsin (a cyclic peptide) [18] and Chidamide (a benzamide-based inhibitor) [19] for the treatment of lymphoma and myeloma. Moreover, several HDACIs such as Mocetinostat [20], Entinostat [21], Tacedinaline [22], Givinostat [23], and Abexinostat [24] are currently in clinical trials for treating various types of cancers. The structures of several approved and clinical HDACIs are shown in Figure 1.

Despite these successes, the most known HDACIs target multiple HDAC isoforms and this poor selectivity represents a major drawback which limits their broad clinical utility [25,26]. Isoform-selective HDACIs would offer superior therapeutic advantages due to limited off-target and undesirable effects, improved clinical efficacy and better tolerability [27,28]. Moreover, isoform-selective inhibitors would provide chemical tools to delineate the precise roles of individual HDAC isoforms in human diseases, including rare disorders [29]. Therefore, in recent years, identification of highly potent inhibitors with strict selectivity towards a specific isoform have caught more attention in the development of novel HDACIs for the epigenetic therapy [30,31,32,33]. In this context, due to the pivotal role of HDAC1 in the angiogenesis, proliferation, and survival of mammalian carcinoma cells, this isoform is particularly being sought as a preferred target for successful design of selective HDACIs [34,35,36,37,38].

A wide range of hydroxamic acid derivatives are known to be potent pan-inhibitors of several HDAC isoforms [39,40]. Accordingly, in recent years, there has been considerable interest in developing non-hydroxamate HDACIs with satisfactory selectivity towards a specific isoform. In this context, aminophenylbenzamide derivatives represent an important class of non-hydroxamate HDACIs owing to their potent HDAC inhibition along with desirable pharmacokinetic profile and excellent selectivity for HDAC class I enzyme. Furthermore, inhibitors containing an ortho-aminobenzamide typically exhibit relatively greater levels of selectivity for class I HDACs, particularly HDAC1 [35,41]. In addition, hydroxamic-derived inhibitors often suffer from some serious pharmacokinetic issues including poor metabolic stability, rapid clearance, undesirable oral absorption, and short half-life in plasma, whereas benzamides-based inhibitors show better metabolic stability and oral bioavailability [42,43,44,45]. For these solid reasons, renewed efforts are being directed towards the further exploration of innovative aminophenylbenzamide chemotypes as privileged and valuable scaffolds to develop isoform-selective HDACIs [46,47,48]. 

Recently, in silico techniques, including ligand-based methods such as pharmacophore modeling and three-dimensional quantitative structural activity relationship (3D-QSAR), have efficiently contributed to guide the discovery of novel bioactive molecules, with reduced costs in terms of money and time [49,50,51]. In fact, QSAR methods provide relationships between physicochemical properties of a series of compounds and their biological activities to obtain a reliable statistical model for predicting the activities of new chemical entities. The fundamental principle of the technique is that the change in structural properties determines modifications in biological activities of the compounds. In the classical QSAR approaches, affinities of ligands to their binding sites, inhibition constants, rate constants, and other biological data have been correlated with molecular properties including lipophilicity, polarizability, electronic and steric properties (Hansch analysis) or with structural features (Free-Wilson analysis). However, classical QSAR approach has only a limited utility for designing new molecules due to the lack of consideration of the 3D structure of the selected compounds. Accordingly, 3D-QSAR has emerged as a natural extension to the classical Hansch and Free-Wilson approaches, which exploits the three-dimensional properties of the ligands to predict their biological activities employing robust chemometric techniques such as partial least squares (PLS). The success of these methods can be attributed to several factors including identification of important features for the activity, rationalization of activity trends in molecules under study, prediction of the specific activity for a selected target or undesirable effects of new compounds. On the other hand, ligand-based methods have been used in virtual screening campaigns of chemical databases to find novel hits with improved potency and can be combined with other computational and experimental workflows to discover new potential drugs [52,53,54,55,56,57].

Until now, only limited number of 3D-QSAR studies for hydroxamate set of HDACIs have been reported [58,59,60,61,62,63,64]. However, to the best of our knowledge, no previous attempt has been made to seek the structural and chemical features of aminophenylbenzamide governing their HDAC inhibitory activities employing 3D-QSAR methodology along with a large set of compounds. Given the aforementioned therapeutic significance of this class of inhibitors, we developed and validated a 3D-QSAR model using a comprehensive set of previously reported benzamide derivatives as selective HDAC1 inhibitors. From this perspective, the software Phase, implemented in Maestro, was employed to explore a common-features pharmacophore hypothesis based on highly active ligands. This hypothesis was then used as an alignment rule to derive a predictive 3D-QSAR model [65]. Such an in silico tool could aid not only in forecasting the HDAC1 inhibitory activity of newly designed chemical entities, but also offer a robust foundation for designing new selective HDACIs with increased binding affinities to HDAC1. Accordingly, the developed model could have a relevant implication in drug discovery campaign for searching isoform-selective HDACIs.

## 2. Results and Discussion

The application of 3D-QSAR methodology in the design of HDACIs has received little attention to date and only a few instances of field-based QSAR models (comparative molecular field analysis (CoMFA) and comparative similarity indices analysis (CoMSIA) methods) have been reported for hydroxamate-based inhibitors [58,59,60,61,62,63,64]. However, this approach has not been carried out for a large set of benzamide-based derivatives behaving as HDACIs. On the other hand, we have recently developed a series of predictive 3D-QSAR models for different purposes including the identification or rational design of new chemical entities for different targets [53,55], and the prediction of undesirable effects of novel molecules such as potential *h*ERG K^+^ channel related cardiotoxicity [57]. In all these cases, Phase was used to develop a computational tool using a pharmacophore-based alignment that links the information of pivotal functional groups of the ligands with their biological activity [65]. The fruitful results of the above-mentioned molecular modeling studies as well as therapeutic significance of benzamide chemotypes as valuable isoform-selective HDACIs inspired us to derive a pharmacophore-based 3D-QSAR model to be used as a screening filtering tool able to quantitatively predict the HDAC1 inhibitory activity of newly designed ligands. 

### 2.1. Data Set Preparation

A comprehensive data set of 370 diverse HDACIs based on benzamide scaffold with functional biological activity expressed as IC_50_ (see the Supplementary Materials for further details) with a range of HDAC1 inhibitory activities spanning five orders of magnitude (from 6.0 nM of compound **17** to 50 µM of compound **132**, Table S1) were selected from literature for developing a predictive 3D-QSAR model. Subsequently, an extensive conformational search for each ligand was performed employing MacroModel software (see experimental section for further details). Conformational analysis is crucial to enhance both the quality of the alignment for the molecules used to generate the 3D-QSAR model and the reliability of the in silico tool [53,54,55,56,57]. After the exhaustive conformational analysis of the selected ligands (Table S1), the generation of the 3D-QSAR model was started.

### 2.2. Pharmacophore Modeling and 3D-QSAR Model Generation 

**Figure 2:**
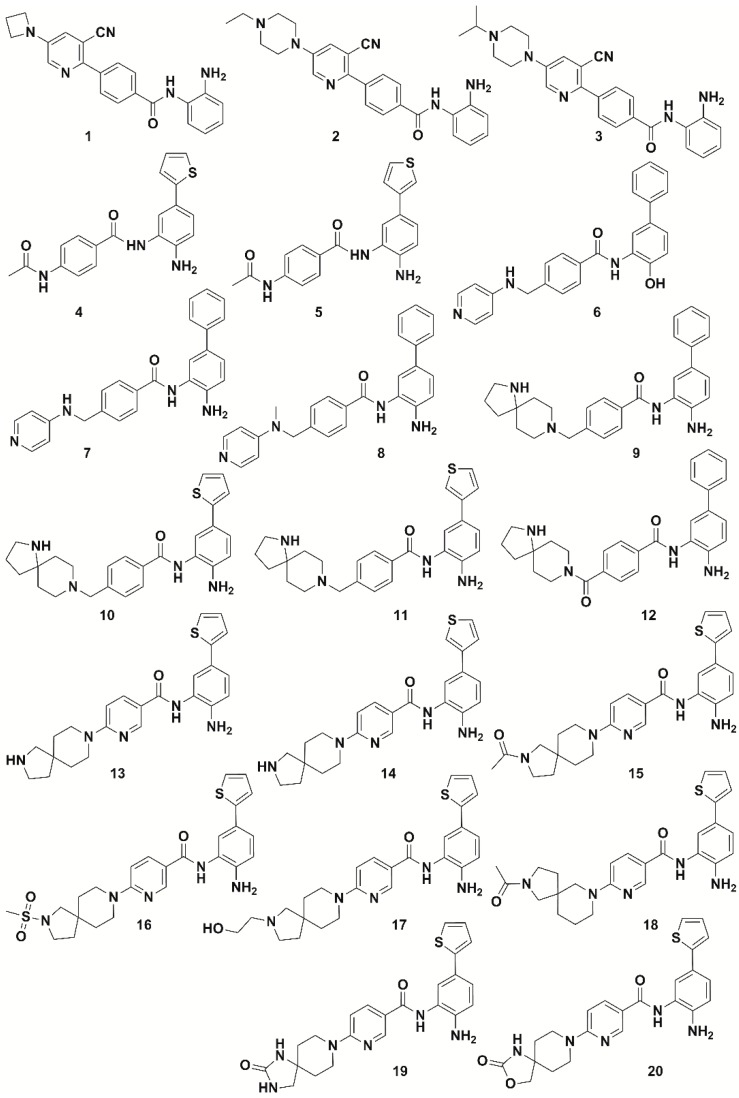
Chemical structure of highly active compounds against HDAC1 (IC_50_ comprised between 0.004 µM and 0.01 µM) used for generating a common-features pharmacophore.

As a first step to develop the 3D-QSAR model, 20 most active compounds with IC_50_ values ≤ 10 nM included in the data set (ligands **1**–**20**, Figure 2, Table S1) were considered to find out common pharmacophore hypotheses that were subsequently scored and ranked by the software Phase. This means that the highly active compounds possess common features that are responsible for the activity exploited by a 3D pharmacophore hypothesis. Therefore, a pharmacophore hypothesis provides a rational picture of primary chemical features of ligands responsible for HDAC1 inhibitory activity and therefore can be used as a reliable alignment rule for the 3D-QSAR model development. 

To have optimal combination of sites or features shared by the most active ligands, the minimum and maximum number of site points were set on 5. This means that we selected 5 as maximum features to include in the pharmacophore models. Among the 26 common pharmacophore hypotheses generated by the software Phase, only those models which showed superior alignment with the active compounds were identified by calculating the survival score. The survival scoring function of Phase module identifies the best candidate hypothesis from the generated models and offers an overall ranking of all the hypotheses. The scoring algorithm includes contributions from the alignment of site points and vectors, volume overlap, selectivity, number of ligands matched, relative conformational energy, and activity. To identify pharmacophore models with more active and less inactive features, all models were mapped to inactive compounds and scored. If inactives score well, the hypothesis could be invalid because it does not discriminate between actives and inactives. Therefore, adjusted survival score was calculated by subtracting the inactive score from the survival score of these pharmacophores.

**Figure 3:**
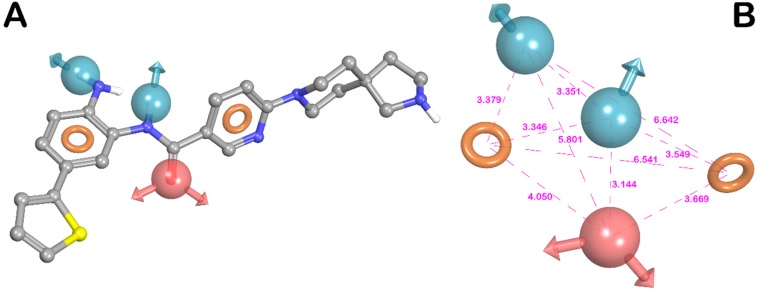
(**A**) Superposition of highly active compound **13** and ADDRR hypothesis. (**B**) ADDRR hypothesis and its inter-feature distances. Features are as follows: H-bond acceptor = A, red vector; H-bond donor = D, blue vectors; aromatic feature = R, orange ring (pictures were generated by means of Maestro software).

After the scoring, the model ADDRR, herein referred to ADDRR hypothesis, with the maximum adjusted survival score (3.769) and lowest relative conformational energy, was selected as the top-ranked hypothesis among the generated 3D model hypotheses. The different scoring parameters for the selected hypothesis (ADDRR) were provided in Table 1. The 3D spatial arrangement of all features with inter-feature distance constraints of ADDRR are presented in Figure 3. As shown in this figure, the hypothesis was characterized by the five main features: one hydrogen-bond acceptor (A), two hydrogen-bond donors (D), and two aromatic rings (R).

Figure 3A depicts one of the most active ligands in the set (compound **13**, Table S1), mapped onto the ADDRR pharmacophore. As depicted in the mentioned figure, compound **13** thoroughly fits all features of the pharmacophore model, underlining the previous findings on structural components required for interacting with the HDAC1 binding site [66,67,68]. As illustrated in Figure 3, the carbonyl oxygen of the benzamide group served as a hydrogen-bond acceptor (HBA) feature, while two hydrogen-bond donor (HBD) features were mapped to the protons of the 2-aminophenyl NH_2_ and amide NH. Furthermore, out of two aromatic features, one was mapped to the phenyl ring of the 2-aminophenyl. The other aromatic feature was mapped to the pyridine ring of the nicotinamide moiety. This hypothesis was well corroborated by accepted common pharmacophore model for HDACIs comprising the zinc binding group (ZBG), a linker and a cap group as established by computational and biophysical studies reported for HDAC1 inhibitors [66,67,68,69,70]. Based on the current study, the aniline groups of the benzamide-based inhibitors are served as ZBG, coordinating the catalytic zinc ion in the HDAC1 active site. Moreover, it is well known that H-bonds formation with key residues of HDAC1 active site are commonly found for the ZBG of this class of HDAC1 inhibitors. In particular, in addition to the zinc ion coordination, protons of the 2-aminophenyl NH_2_ group could also establish hydrogen bonds with His140 and His141, while the carbonyl oxygen of the benzamide portion could also form another hydrogen binding interaction with hydroxyl group of Tyr303. It has been reported that NH of amide could offer the appropriate HBD vector to address the Gly149 through H-bond formation. The presence of two aromatic features capable of participating in π-π stacking interactions with hydrophobic residues Phe150, Tyr204, Phe205, and Tyr303, represents another important requisite for further stabilization of ligand binding. These residues were located in a long and narrow hydrophobic tube-like channel and thus interaction with them allow tubular access of ligand into active site [66,67,69,70]. Accordingly, the above-mentioned ADDRR hypothesis imparts the key features of ligand for providing the relevant interactions with the HDAC1 active site.

The ADDRR pharmacophore hypothesis was then employed as alignment rule to derive the 3D-QSAR model. In this step, the compounds were randomly divided into training (70%) and test sets (30%) taking into account that the response range was well-covered in both sets (Table S1). This choice was made to warrant the inclusion of the positive information originating from 70% of the compounds enclosed in the training set (corresponding to 259 compounds), for the development of the computational tool. Moreover, the compounds kept in the test set (30%, 111 compounds) ensures an appropriate assessment of the predictive power of the generated model through an exhaustive internal validation. The atom-based version of Phase’s 3D-QSAR workflow was preferred to the pharmacophore-based one. Such a choice allowed us to take into account contributions associated with all the important structural features other than pharmacophore for HDAC1 inhibitory activity such as the steric clashes. To enhance the model accuracy and evade overfitting phenomenon, models containing one up to seven factors were generated for the studied data set. Statistical parameters for each model are provided in Table 2. Model featuring seven factors was preferred and selected because it better performed in comparison with other models. The reliability of the selected model is justified by the fact that all statistical parameters were in acceptable range. In this regard, the correlation and cross-validated correlation coefficients (R^2^ = 0.958 and Q^2^ = 0.822, respectively) of the selected model along with the Pearson R-value (R-Pearson = 0.915) were extremely satisfactory, indicating a close correspondence between estimated and experimental IC_50_ values. Moreover, the high Fisher ratio (F = 822.1) suggested a statistically significant regression model, which was further supported by the small value of the variance ratio (P = 4.377 × 10^−169^), an indication of a high degree of confidence. Finally, the small values of the standard deviation and the root-mean-square error (SD: 0.178 and RMSE: 0.281, respectively) also provided indication about the robustness of the developed computational model. Moreover, the Q^2^_F3_ value clearly indicates that the 3D-QSAR model with seven factors is robust. 

**Figure 4:**
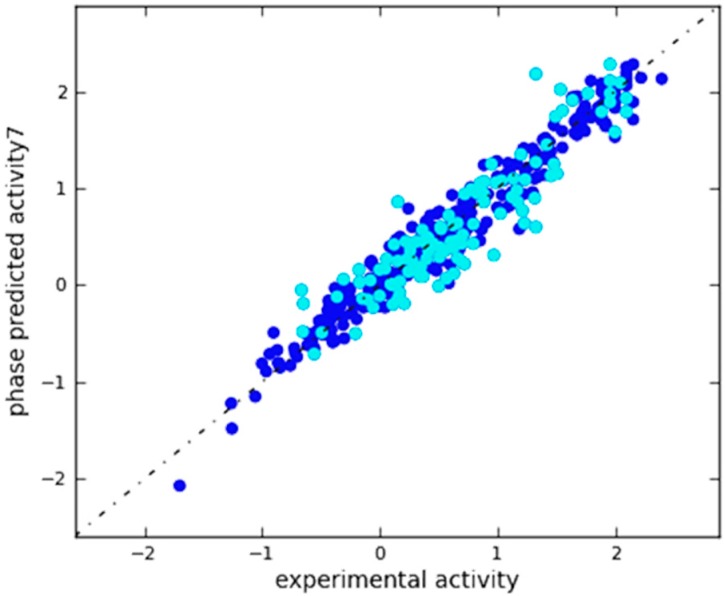
Scatter plot for the predicted (Phase-predicted activity) and the observed (experimental activity) pIC_50_ values (μM) as calculated by the 3D-QSAR model applied to the training set (blue) and test set (cyan) compounds.

A scatter plot of experimental versus predicted activities was generated to assess the results (Figure 4). Based on this plot, the IC_50_ values were reliably predicted for both training and test set molecules (Table S1). This plot along with the aforementioned statistical features clearly imply the significance of the approach and indicate a QSAR model with a robust predictive power.

**Figure 5:**
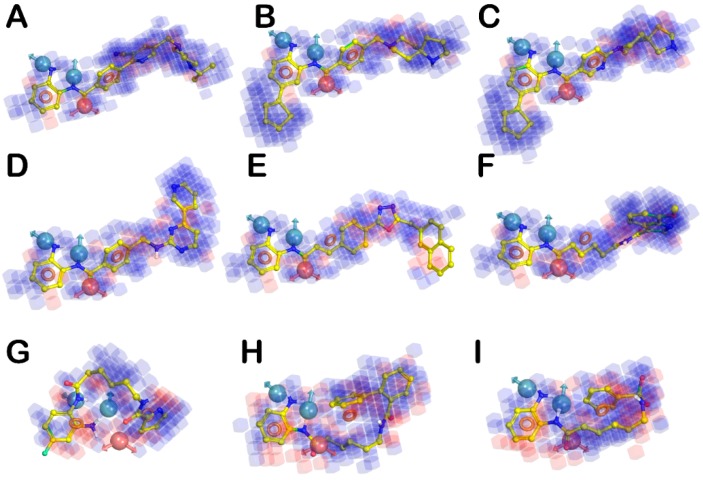
(**A**–**C**) Superposition of highly active compounds **3**, **10**, and **13**, respectively with the 3D-QSAR model. (**D**–**F**) Superposition of moderate active compounds **22** (Mocetinostat), **213**, and **239**, respectively with the 3D-QSAR model. (**G**–**I**) Superposition of less active compounds **218**, **279**, and **299**, respectively with the 3D-QSAR model. The picture was generated by means of Maestro software (Schrödinger, LLC, New York, NY, USA, 2015).

Three-dimensional aspects obtained from the QSAR model were visualized using 3D plots of the crucial volume elements occupied by ligands. Such plots allow the visual analysis of important features of ligand structures along with their contributions to the biological activity. The 3D plot representation of the whole model, superimposed to the highly (**3, 10**, and **13**), moderate (**22, 213,** and **239**), and less active derivatives (**218, 279**, and **299**), is depicted in Figure 5. In this illustration, the blue and red cubes indicate the positive and negative coefficients, respectively. In fact, blue cubes refer to ligand regions in which the specific feature is important for better activity, whereas the red cubes are indicative of a particular structural feature or functional group which is not essential for the activity or is likely to decrease the activity. Cubes with small positive and negative coefficients, which therefore did not greatly affect the activity, were filtered out by setting a 1.50 × 10^−2^ coefficient threshold. Remarkably, compounds **10** and **13** (Figure 5A,B, respectively) as well as other highly active ligands, mainly lodge in the blue regions, while the less active compounds such as **218** and **299** (Figure 5G,I, respectively) largely resides on the red regions. Moreover, regarding some compounds with moderate activity and generally all compounds with limited activity, we also observed a significant inability to match all the pharmacophore features, according to the decrease of inhibitory potencies.

### 2.3. In Silico 3D-QSAR Model Validation

#### 2.3.1. Validation Using External Test Set

**Figure 6:**
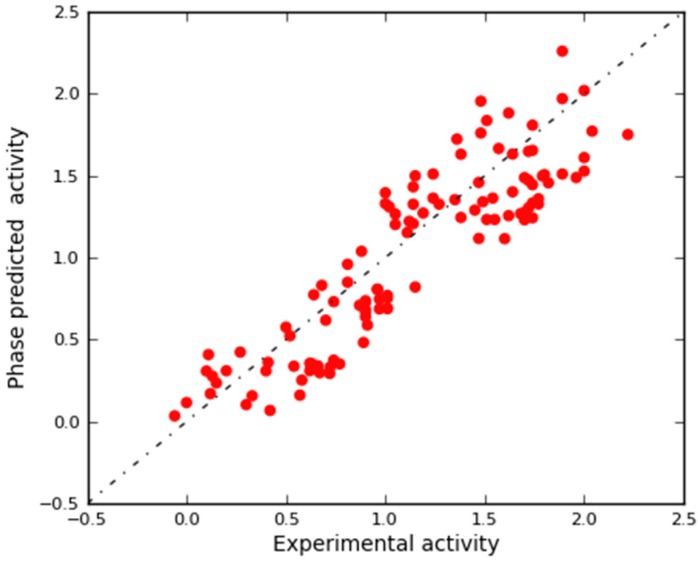
Scatter plot for the predicted (Phase-predicted activity) and observed (experimental activity) pIC_50_ values (μM) as calculated by the 3D-QSAR model with 7 factors applied to the external test set.

After the generation of the 3D-QSAR model, a preliminary in silico validation was performed using an external test set selected from the literature that have not been used for generating the computational model. This set was composed of 113 compounds with different inhibitory activities against HDAC1 (ranging from 5.8 nM to 1140 nM; Table S2 in the Supplementary Materials). As reported in Table S2, our model was satisfactorily efficient in estimating the HDAC1 inhibitory activity of compounds included in the external test set. In the scatter plot depicted in Figure 6, the experimental and predicted pIC_50_ values of these compounds are also displayed, offering a reasonable correlation coefficient (r^2^_ext_ts_ = 0.794). This result provided further confirmation that the correlation shown by the model is not accidental.

#### 2.3.2. Validation Using Decoy Set and Receiver-Operating Characteristic (ROC) Curve Approach

**Figure 7:**
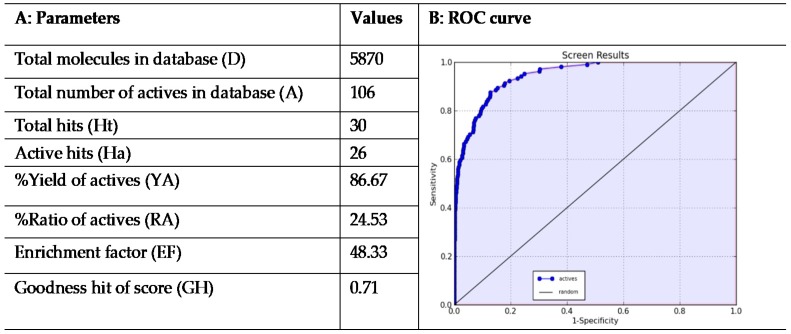
(**A**) EF and GH scores obtained by the application of 3D-QSAR model in a database screening; and (**B**) receiver-operating characteristic (ROC) curve generated from database screening.

For a further validation and to assess the performance of the developed 3D-QSAR model, we employed a validation method based on generation of decoys set. This procedure is usually employed to evaluate the capability of in silico tools such as 3D-QSAR models to discriminate between active or inactive molecules [71,72,73,74,75]. Starting from highly active compounds (ligands 1–20 in Table S1), 86 additional compounds with good activity against HDAC1 (cutoff IC_50_ < 35 nM; Tables S1 and S2) were selected from the training, test and external validation sets for a total of 106 compounds (Table S3) from which decoys were generated. For this set of active ligands, DUD-E server generated 5764 decoys. After an appropriate minimization and conformational search of decoys, we have combined them with the active molecules (referred as A in Figure 7A) for a total of 5870 compounds (referred as D in Figure 7A) that were then subjected to a virtual screening using the developed 3D-QSAR model. Interestingly, the results of this evaluation supported the validity of the proposed model. Analysis of the database screening results (Figure 7A) indicated a trend in which inactive compounds fail to completely satisfy all the pharmacophore features, thus making their predicted activity very poor or absent. In contrast, the 3D-QSAR model was reasonably efficient in the estimation of HDAC1 inhibitory activity of active compounds.

According to the screening results (Figure 7A), the top 30 ranked compounds were considered to be hits (Ht). This cutoff value could represent a suitable number of molecules (about 1% of database) to be purchased after a virtual screening campaign. Remarkably, among Ht, 26 (Ha) compounds belonged to the set of 106 known HDAC1 inhibitors. Furthermore, this qualitative analysis was well supported by the calculation of some statistical parameters such as EF and GH score (see Materials and Methods section for calculation details). In this regard, the calculated EF was 48.33, which implies that it could be about 48.33 times more probable to select active compounds from the hit list compared with random selection from the complete database. The estimated GH score value of 0.71, larger than 0.5, indicates a great reliability of the model (Figure 7A). This suggests that the developed computational model can serve as efficient tool in virtual screening studies to find out novel chemical entities behaving as selective HDAC1 inhibitors.

The applicability of the proposed 3D-QSAR model was further evaluated by means of the receiver-operating characteristic (ROC) curve. The ROC curve approach is a well-recognized metric used as an objective way to assess the balance between model sensitivity (capability to discover true positives) and specificity (capability to avoid false positives) [55,57,76,77]. For this purpose, 5870 compounds employed in the previous validation step, were ranked according to their predicted activity values as estimated by the 3D-QSAR model. The output of the ROC curve provided a score for appraising the overall performance of the model. In particular, the closer the ROC score is to 1.0, the better is the model at discriminating active from inactive compounds. ROC curve analysis of our in silico model yielded a satisfactory Area Under the Curve (AUC) score of 0.94 (Figure 7B), providing additional evidence about the predictivity of the developed 3D-QSAR model.

## 3. Materials and Methods 

### 3.1. Hardware and Software Specifications

All computational tasks in this study were carried out using molecular modeling package from Schrödinger suite 2015 (Schrödinger, Inc., LLC, New York, NY, USA) installed on an Intel(R) Xeon(R) CPU E5-2620 v2 @ 3.30 GHz, 64 GB RAM with 12 processors, and a 2GB graphics card of NVIDIA Quadro K2200 running Ubuntu 10.04 LTS (long-term support) as operating system. Access to the Schrödinger modules as well as the capability to organize and analyze data was provided by Maestro as a portal interface of Schrödinger [78].

### 3.2. Ligands and Data Set Preparation

A comprehensive set of HDAC1 inhibitors characterized by the 2-aminophenylbenzamide scaffold with known IC_50_ values that vary over a wide range was collected from the literature [66,67,79,80,81,82,83,84,85,86,87,88,89,90,91,92] and the bindingDB database [93]. The selection criterion for the compounds to be included in the set was that their HDAC1 inhibition was evaluated using the same fluorescent assay based on the fluorogenic substrate Fluor-de-Lys. This inclusion criterion allowed us to obtain a homogeneous set of compounds regarding their biological evaluation. This step is crucial to develop a predictive model since the data selection is pivotal for adding the correct information to a software for developing computational models. The 3D structures of all ligands were built using the builder panel in the Maestro. For the molecules possessing known stereochemistry, the absolute configuration was specified during the drawing of the compounds. All structures were treated by LigPrep module of Schrödinger suite 2015 [94] in order to generate the most probable ionization state at the cellular pH value (7.4 ± 0.2) as reported by us [95,96,97]. Moreover, the OPLS-AA_2005 force field was used for optimization, which produces the lowest energy conformer of the ligand [98]. The prepared ligands were then submitted to MacroModel software [99] in order to obtain an exhaustive conformational analysis using the OPLS-AA_2005 as force field. The solvent effects are simulated employing the analytical Generalized-Born/Surface-Area (GB/SA) model [100], and no cutoff for non-bonded interactions was selected. Molecular energy minimizations were performed using Polak–Ribiere conjugate gradient (PRCG) method with 2000 maximum iterations and 0.001 gradient convergence threshold. The conformational searches were carried out by employing MCMM (Monte Carlo Multiple Minimum) torsional sampling method. Automatic setup with 21 kJ/mol (5.02 kcal/mol) in the energy window for saving structure and a 0.5 Å cutoff distance for redundant conformers was used.

### 3.3. 3D-QSAR Model Generation

The software package Phase 4.2 [101], implemented in Maestro suite, was used to generate pharmacophore hypotheses and 3D-QSAR models for HDAC1 inhibitors based on 2-aminophenylbenzamide scaffold. Given a set of molecules with high affinity for a particular protein target, this software uses fine-grained conformational sampling and a range of scoring techniques to identify a common-features pharmacophore hypothesis, which conveys 3D structural characteristics that are critical for the activity. Pharmacophore feature sites for the molecules were specified by a set of features well-defined in Phase as hydrogen-bond acceptor (A), hydrogen-bond donor (D), hydrophobic group (H), negatively charged group (N), positively charged group (P) and aromatic ring (R). No user-defined feature was employed for the present study. The ligands prepared as reported in the previous step, were imported into the “develop common pharmacophore hypotheses” panel of Phase with their respective biological activity values. Twenty active compounds (Figure 2 and in Table S1 in the Supplementary Materials) possessing highly inhibitory potency against HDAC1 were selected for generating the pharmacophore hypotheses. Common-features pharmacophore hypotheses were identified, scored, and ranked by means of conformational analysis and tree-based partitioning techniques. In the score hypotheses step, common pharmacophores are examined, and a scoring procedure is applied to identify the pharmacophore from each surviving n-dimensional box that yields the best alignment of the active set ligands. This pharmacophore provides the means to explain how the active molecules bind to the receptor.

The best ranked pharmacophore model obtained by Phase (ADDRR, shown in Figure 3A superimposed to aminophenylnicotinamide analogue **13**), consisted of five features: one hydrogen-bond acceptor (A), two hydrogen-bond donors (D), and two aromatic functions (R). The inter-feature distances (Figure 3B) were measured by using the site measurements tool implemented in the software Phase. This pharmacophore was used as alignment rule for further 3D-QSAR analysis. All the molecules used for the QSAR studies (Table S1) were aligned to the selected pharmacophore hypothesis. In the present study, we set a pIC_50_ threshold for the selection of active and inactive ligands. These pIC_50_ values were also used as the dependent variable in the 3D-QSAR calculations. In particular, compounds that showed an IC_50_ comprised between 5 and 50 µM were considered to be inactive ligands. Moderate inhibitors were considered compounds with IC_50_ between 10 nM and 5 µM, while compounds possessing an IC_50_ ≤ 10 nM were assigned as potent inhibitors of HDAC1 and consequently as actives during 3D-QSAR model generation. Remarkably, to avoid possible faults arising from the inclusion in the set of molecules with uncertain activity, only molecules with experimentally definite inhibitory activity have been selected to develop the in silico model. Atom-based QSAR models were developed for ADDRR hypothesis using 259 compounds in the training set (370 compounds were randomly divided 70% in the training and 30% in the test set) and a grid spacing of 0.5 Å. QSAR models were generated by means of PLS method. An internal validation was achieved employing leave-n-out (LnO) technique as specified in Phase user manual (Phase, version 4.2, User Manual, Schrödinger press, LLC, New York, NY, 2015). As reported by Todeschini et al. the internal validation results generally expressed in terms of Q^2^ metrics should be amended introducing Q^2^_F3_ metrics for the internal validation of the QSAR models [102]. For this purpose, we calculated these metrics (Table 2) employing the formula reported below (Equation (1)).

(1) $$Q_{F3}^{2}=1-\frac{\sum_{i=1}^{n_{OUT}}{\left(y_{i}-{\hat{y}}_{i/i}\right)}^{2}/n_{OUT}}{\sum_{i=1}^{n_{TR}}{\left(y_{i}-{\bar{y}}_{TR}\right)}^{2}/n_{TR}}\;$$

where *y_i_* is the experimental response of the ith object, *ŷ_i/i_* is the predicted response when the ith object is not in the training set, *n_TR_* and *n_OUT_* are the number of training and prediction objects, respectively, and *ȳ_TR_* is the average value of the training set experimental responses. Moreover, to avoid overfitting/underfitting phenomena, we considered 7 factors that is an appropriate for the number of selected compounds. In fact, although there is no limit on the maximum number of factors, but as a general rule, we stopped adding factors when the standard deviation of regression is approximately equal to the experimental error (calculated as median error among the selected compounds).

### 3.4. In Silico 3D-QSAR Model Validation

After the generation of the 3D-QSAR model, a preliminary in silico validation was performed using a large external test set of compounds (113 molecules) selected from the literature [83,84,89,103,104,105,106] (Table S2 in the Supplementary Materials) that have not been used for generating and cross validating the model. These compounds were prepared by using Maestro, LigPrep, and MacroModel, adopting the same procedure for preparing the molecules used to derive the model. Moreover, to further assess that the chosen model with 7 factors better performs with respect to the other Phase-derived models, we applied the validation method employing the external test set to all the generated QSAR models (Table 2). This workflow established that the model with 7 factors is the best performing model of the series in predicting the activity of the external test set with a correlation coefficient r^2^_ext_ts_ = 0.794 (Figure 6) (LVs 1, r^2^_ext_ts_ = 0.421; LVs 2, r^2^_ext_ts_ = 0.698; LVs 3, r^2^_ext_ts_ = 0.657; LVs 4, r^2^_ext_ts_ = 0.712; LVs 5, r^2^_ext_ts_ = 0.735; LVs 6, r^2^_ext_ts_ = 0.787; Figures S1–S6, respectively).

Further validation of the model was done by enrichment study using decoy test [107]. For this purpose, the Enhanced (DUD-E) web server [108] was employed to generate a set of useful decoys generated from a collection of 106 active compounds from three sources: 1) active compounds used to develop the pharmacophore model, 2) other compounds with good activity against HDAC1 used in 3D-QSAR studies and 3) the most active compounds of the external test set. This collection consisted of 106 active compounds with IC_50_ ≤ 35 nM (Table S3). For this set of active ligands, the DUD-E server provided 5764 inactive ligands (redundant structures in the output files were deleted) from a subset of the ZINC database filtered using the Lipinski’s rules for drug-likeness, for a total of 5870 compounds (5764 inactives plus 106 actives). Each of these inactive decoys was selected to bear a resemblance to the physicochemical properties of the reference ligand but differ from it in terms of 2D structure (e.g., large difference of Tanimoto coefficient between decoys and active molecules). Although largely used, the approach based on decoys sets presents some limitations (i.e., the decoy sets often span a small, synthetically feasible subset of molecular space and are restricted in physicochemical similarity compared with actives). After the generation, the decoys sets were downloaded as 126 smiles files and imported into Maestro and submitted to LigPrep application to properly convert smiles into 3D structures as well as for removing potential erroneous structures. Subsequently, to perform a minimization and a conformational search of the obtained structures MacroModel program was employed (same parameters for ligands preparation were applied). A single file containing conformers of active molecules and decoys was created and submitted to Phase software for predicting the inhibitory activity of database against HDAC1 using the developed 3D-QSAR model and employing “search for matches” option. After decoys generation and activity evaluation, the Güner–Henry score, i.e., goodness of hit list (GH) and enrichment factor (EF) values were estimated by Equations (2) and (3), respectively.

(2) $$EF=\frac{Ha/Ht}{\left(A/D\right)}\;$$

(3) $$GH=\left\{\frac{Ha*\left(3A+Ht\right)}{4HtA}\right\}*\left[1-\frac{\left(Ht-Ha\right)}{\left(D-A\right)}\right]$$

where *Ht* represents the total number of compounds in the hit list found by virtual screening, *Ha* is the total actives found by virtual screening considering the top 30-ranked position (positions comprise within the cutoff value). The total number of compounds (*Ht*) might represent the number of molecules to purchase after a virtual screening protocol and almost the 1% of the considered database (*D*). *A* represents the total of the active derivatives enclosed in the database, and *D* stands for the total number of molecules existing in the set. The range of *GH* score varies from 0 to 1. The *GH* score 0 means a null model, while the *GH* score 1 denotes generation of an ideal model. Moreover, the % yield of actives (% *YA*) and % ratio of actives (% *RA*) were evaluated by Equations (4) and (5), respectively.

(4) $$\%YA=\left[\left(\frac{Ha}{Ht}\right)*100\right]\;$$

(5) $$\%RA=\left[\left(\frac{Ha}{A}\right)*100\right]\;$$

Moreover, to assess the predictive power of the 3D-QSAR model, a ROC was employed through an Enrichment Calculator (enrichment.py) script [55,56,57,76]. The mentioned script calculates the enrichment metrics, including area under the receiver-operating characteristic curve (AUC), from virtual screening by means of the output structure file and a list of known active molecules. The output of the screening protocol, using active molecules and decoys, consisted of a list of molecules ranked by the predicted activity from the top-predicted molecules as estimated by the 3D-QSAR model. These ranking data along with a list file of active molecules were submitted to the enrichment.py application.

## 4. Conclusions

The present study describes the generation of a ligand-based pharmacophore model (ADDRR) for a subset of 20 highly active aminophenylbenzamide derivatives reported as selective HDAC1 inhibitors by employing the software Phase implemented in the Schrödinger molecular modeling suite. With the aid of pharmacophore-based alignment rule, a meaningful 3D-QSAR model was derived and validated employing of the QSAR models a large set of benzamide-based HDAC1 inhibitors (training set, test set, and an external test set for a total of 483 molecules) by using PLS analysis. The main objective of this approach was to develop an in-house computational tool for the prediction of HDAC1 inhibitory activity during the design of innovative aminophenylbenzamide chemotypes as privileged therapeutic scaffold in the isoform-selective HDACIs research. The validation outcomes confirmed that the proposed 3D-QSAR model is endowed with satisfactory predictive power taking into account favorable structural requirements responsible for HDAC1 inhibitory activity. This aspect has been computationally investigated since the selectivity is implicit in the template molecules; however prospective validation is needed to exploit the performance of the model. In fact, the developed 3D-QSAR model can be used for rationally designing novel and selective HDACIs. Moreover, based on the computational investigation, the developed model possesses a rationale for virtual screening campaign, with huge potential in isoform-selective HDACIs drug discovery, and it can effectively provide a set of guidelines for the design and optimization of novel derivatives with greater activity towards HDAC1.

## Figures and Tables

**Table 1:** The different scoring parameters for the best pharmacophore hypothesis, matching all 20 highly active compounds used.

HYPO ID	Survival	Survival—Inactive	Site	Vector	Volume	Selectivity	Matches	Energy	Activity	Inactive
**ADDRR**	3.769	1.841	0.97	0.999	0.798	1.578	20	0.006	2.097	1.928

**Table 2:** 3D-QSAR statistical parameters of the seven Latent Variables (LVs) Phase-derived sets of models.

LVs	R ^2a^	SD ^b^	F ^c^	P ^d^	RMSE ^e^	Q ^2f^	Q^2^_F3_^g^	R-Pearson ^h^
1	0.3408	0.6978	132.9	4.699 × 10^−22^	0.5199	0.3886	0.635	0.6747
2	0.6273	0.5257	215.5	1.344 × 10^−55^	0.3979	0.6420	0.791	0.8282
3	0.7620	0.4209	272.2	3.629 × 10^−79^	0.3615	0.7045	0.823	0.8579
4	0.8775	0.3025	455.0	1.704 × 10^−144^	0.3514	0.7207	0.833	0.8690
5	0.9159	0.2512	551.0	9.327 × 10^−134^	0.2971	0.8004	0.881	0.9003
6	0.9433	0.2067	698.6	6.590 × 10^−134^	0.2865	0.8143	0.890	0.9100
7	0.9582	0.1778	822.1	4.377 × 10^−169^	0.2808	0.8217	0.894	0.9152

^a^R^2^: value of r^2^ of the regression. ^b^SD: standard deviation of the regression. ^c^F: variance ratio. ^d^P: significance level of variance ratio. ^e^RMSE: root-mean-square error in the test set predictions. ^f^Q^2^: value of Q^2^ for the predicted activities. ^g^Q^2^_F3_: value of Q^2^_F3_^:^ for the predicted activities calculated as reported in Materials and Methods section. ^h^R-Pearson: correlation between the predicted and observed selectivity index values for the test set.

## Supplementary Materials

The following are available online, Table S1: Experimental (Observed column) and predicted (Predicted column) activity pIC_50_ for compounds used for developing the 3D-QSAR model., Table S2: Experimental (Observed column) and predicted (Predicted column) activity pIC_50_ for compounds included in the external test set, Table S3: Active compounds used for generating a decoys set, Figures S1–S6: Scatter plots for all the model generated by Phase.
