# Supplementary material for: Computer-Driven Development of an in Silico Tool for Finding Selective Histone Deacetylase 1 Inhibitors
Source: Molecules. 2020 Apr 22;25(8):1952. doi: 10.3390/molecules25081952 (PMC7221830; doi:10.3390/molecules25081952)
Supplement: Supplementary file 1 [file molecules-25-01952-s001.pdf]

molecules

Supplementary Materials

Computer-Driven Development of an in Silico Tool for Finding Selective Histone Deacetylase 1 Inhibitors

Hajar Sirous [1,*], Giuseppe Campiani [2], Simone Brogi [3,*], Vincenzo Calderone [3] and Giulia Chemi [2,†]

[1] Bioinformatics Research Center, School of Pharmacy and Pharmaceutical Sciences, Isfahan University of Medical Sciences, 81746-73461, Isfahan, Iran
[2] Department of Biotechnology, Chemistry and Pharmacy, DoE Department of Excellence 2018-2022, University of Siena, via Aldo Moro 2, 53100, Siena, Italy; campiani@unisi.it (G.C.); GChemi001@dundee.ac.uk (G.Ch.)
[3] Department of Pharmacy, University of Pisa, via Bonanno 6, 56126, Pisa, Italy; vincenzo.calderone@unipi.it
* Correspondence: h_sirous@pharm.mui.ac.ir (H.S.); simone.brogi@unipi.it (S.B.); Tel.: +98-313-792-7065 (H.S.); +39-050-2219613 (S.B.)
† Present address: Wellcome Centre for Anti-Infectives Research, Drug Discovery Unit, Division of Biological Chemistry and Drug Discovery, University of Dundee, DD1 5EH Dundee, UK.

Table of Contents

Table S1 **page S2**

Table S2 **page S21**

Table S3 **page S27**

Figure S1-S6 **page S30**

References **page S32**

Table S1. Experimental (Observed column) and predicted (Predicted column) activity pIC_{50} for compounds used for developing the 3D-QSAR model. (Note: a) compounds included in the training set; b) compounds included in the tests set; (Note: * compounds used for the decoys generation).

	Compounds	Observed IC_{50}(μM)	Observed PIC_{50}	Predicted pIC_{50}	Note
**1**	C1CCN1Cc(cn2)cc(C#N)c2-c3ccc(cc3)C(=O)Nc4c(N)cccc4 (Andrews and Gibson, et al., 2008)	0.01	2.000	1.830	a*
**2**	c1cccc(N)c1NC(=O)c(cc2)ccc2-c3c(C#N)cc(cn3)CN4CCN(CC4)CC (Andrews and Gibson, et al., 2008)	0.01	2.000	1.880	a*
**3**	c1cccc(N)c1NC(=O)c(cc2)ccc2-c3c(C#N)cc(cn3)CN4CCN(CC4)C(C)C (Andrews and Gibson, et al., 2008)	0.01	2.000	1.970	a*
**4**	c1ccsc1-c(ccc2N)cc2NC(=O)c3ccc(cc3)NC(=O)C (Methot and Chakravarty, et al., 2008)	0.007	2.155	1.710	a*
**5**	c1cscc1-c(ccc2N)cc2NC(=O)c3ccc(cc3)NC(=O)C (Methot and Chakravarty, et al., 2008)	0.007	2.155	1.890	a*
**6**	c1ccccc1-c(c2)ccc(O)c2NC(=O)c3ccc(cc3)CNc4ccncc4 (Kattar, et al., 2009)	0.01	2.000	1.530	a*
**7**	c1ccccc1-c(c2)ccc(N)c2NC(=O)c3ccc(cc3)CNc4ccncc4 (Kattar, et al., 2009)	0.01	2.000	1.580	b*
**8**	c1ccccc1-c(c2)ccc(N)c2NC(=O)c3ccc(cc3)CN(C)c4ccncc4 (Kattar, et al., 2009)	0.01	2.000	2.020	a*
**9**	c1ccccc1-c(c2)ccc(N)c2NC(=O)c3ccc(cc3)CN(CC4)CCC45NCCC5 (Kattar, et al., 2009)	0.008	2.097	1.790	b*
**10**	s1cccc1-c(c2)ccc(N)c2NC(=O)c3ccc(cc3)CN(CC4)CCC45NCCC5 (Kattar, et al., 2009)	0.004	2.398	2.130	a*
**11**	c1sccc1-c(c2)ccc(N)c2NC(=O)c3ccc(cc3)CN(CC4)CCC45NCCC5 (Kattar, et al., 2009)	0.008	2.097	2.250	a*
**12**	c1ccccc1-c(c2)ccc(N)c2NC(=O)c3ccc(cc3)C(=O)N(CC4)CCC45NCCC5 (Kattar, et al., 2009)	0.008	2.097	2.070	a*
**13**	C1CNCC12CCN(CC2)c(nc3)ccc3C(=O)Nc4cc(ccc4N)-c5cccs5 (Methot and Hamblett, et al., 2008)	0.008	2.097	1.930	b*
**14**	C1CNCC12CCN(CC2)c(nc3)ccc3C(=O)Nc4cc(ccc4N)-c5ccsc5 (Methot and Hamblett, et al., 2008)	0.01	2.000	2.060	a*
**15**	C1CN(C(=O)C)CC12CCN(CC2)c(nc3)ccc3C(=O)Nc4cc(ccc4N)-c5cccs5 (Methot and Hamblett, et al., 2008)	0.009	2.046	2.090	b*
**16**	C1CN(S(=O)(=O)C)CC12CCN(CC2)c(nc3)ccc3C(=O)Nc4cc(ccc4N)-c5cccs5 (Methot and Hamblett, et al., 2008)	0.008	2.097	2.190	a*
**17**	OCCN(CC1)CC12CCN(CC2)c(nc3)ccc3C(=O)Nc4cc(ccc4N)-c5cccs5 (Methot and Hamblett, et al., 2008)	0.006	2.222	2.140	a*
**18**	CC(=O)N(C1)CCC12CCCN(C2)c(nc3)ccc3C(=O)Nc4cc(ccc4N)-c5cccs5	0.008	2.097	2.060	a*

	(Methot and Hamblett, et al., 2008)				
**19**	C1NC(=O)NC12CCN(CC2)c(nc3)ccc3C(=O)Nc4cc(ccc4N)-c5cccs5<br>(Methot and Hamblett, et al., 2008)	0.007	2.155	2.280	a*
**20**	C1OC(=O)NC12CCN(CC2)c(nc3)ccc3C(=O)Nc4cc(ccc4N)-c5cccs5<br>(Methot and Hamblett, et al., 2008)	0.008	2.097	2.130	a*
21	Nc1ccccc1NC(=O)c2ccc(cc2)CNC(=O)OCc3cccnc3<br>**Entinostat**<br>(Li et al., 2015)	0.273	0.564	0.410	a
22	Nc1ccccc1NC(=O)c2ccc(cc2)CNc3nccc(n3)-c4cccnc4<br>**Mocetinostat**<br>(Li et al., 2013)	0.098	1.01	0.800	a
**23**	Nc1ccccc1NC(=O)c2ccc(cc2)C(C(=O)Nc3ccccc3)C(=O)Nc4ccccc4<br>(Siliphaivanh et al.,2007)	0.041	1.387	1.320	a
**24**	Nc1ccccc1NC(=O)c2ccc(cc2)C(C(=O)Nc3cccc(c3)C#N)C(=O)Nc4cc(C#N<br>)ccc4<br>(Siliphaivanh et al.,2007)	0.034	1.469	1.280	a*
**25**	Nc1ccccc1NC(=O)c2ccc(cc2)C(C(=O)Nc3ccc(F)cc3)C(=O)Nc4ccc(F)cc4<br>(Siliphaivanh et al.,2007)	0.063	1.201	1.350	b
**26**	Nc1ccccc1NC(=O)c2ccc(cc2)C(C(=O)Nc3cc(F)ccc3)C(=O)Nc4cc(F)ccc4<br>(Siliphaivanh et al.,2007)	0.056	1.252	1.210	a
**27**	Nc1ccccc1NC(=O)c2ccc(cc2)C(C(=O)Nc3cc(OC)ccc3)C(=O)Nc4cc(OC)cc<br>c4<br>(Siliphaivanh et al.,2007)	0.035	1.456	1.380	a*
**28**	Nc1ccccc1NC(=O)c2ccc(cc2)C(C(=O)Nc3c(OC)cccc3)C(=O)Nc4ccccc4OC<br>(Siliphaivanh et al.,2007)	0.047	1.328	1.320	a
**29**	Nc1ccccc1NC(=O)c2ccc(cc2)C(C(=O)Nc(cc3)cc(c34)OCCO4)C(=O)Nc(c5<br>)ccc(c56)OCCO6<br>(Siliphaivanh et al.,2007)	0.021	1.678	1.550	a*
**30**	Nc1ccccc1NC(=O)c2ccc(cc2)C(C(=O)Nc3cc(ccc3)N4CCOCC4)C(=O)Nc5c<br>ccc(c5)N6CCOCC6<br>(Siliphaivanh et al.,2007)	0.045	1.347	1.280	a
**31**	Nc1ccccc1NC(=O)c2ccc(cc2)C(C(=O)Nc3cccc(c34)cccn4)C(=O)Nc5cccc(<br>c56)cccn6<br>(Siliphaivanh et al.,2007)	0.103	0.987	1.060	b
**32**	Nc1ccccc1NC(=O)c2ccc(cc2)C(C(=O)N(C)c3ccccc3)C(=O)Nc4ccccc4<br>(Siliphaivanh et al.,2007)	0.196	0.708	0.790	a
**33**	Nc1ccccc1NC(=O)c2ccc(cc2)C(C(=O)N(C)c3ccccc3)C(=O)N(C)c4ccccc4<br>(Siliphaivanh et al.,2007)	7.195	-0.857	-0.810	a
**34**	Nc1ccccc1NC(=O)c2ccc(cc2)C(C(=O)NCc3ccccc3)C(=O)NCc4ccccc4<br>(Siliphaivanh et al.,2007)	0.199	0.701	0.630	a

**35**	Nc1ccccc1NC(=O)c2ccc(cc2)C(C(=O)NCCCc3ccccc3)C(=O)NCCCc4ccccc4 (Siliphaivanh et al.,2007)	0.229	0.640	0.690	a
**36**	Nc1ccccc1NC(=O)c2ccc(cc2)C(F)(C(=O)Nc3ccccc3)C(=O)Nc4ccccc4 (Siliphaivanh et al.,2007)	0.048	1.319	1.100	a
**37**	Nc1ccccc1NC(=O)c2ccc(cc2)C(O)(C(=O)Nc3ccccc3)C(=O)Nc4ccccc4 (Siliphaivanh et al.,2007)	0.094	1.027	0.740	b
**38**	Nc1ccccc1NC(=O)c2ccc(cc2)C(C)(C(=O)Nc3ccccc3)C(=O)Nc4ccccc4 (Siliphaivanh et al.,2007)	0.198	0.703	0.660	a
**39**	Nc1ccccc1NC(=O)c2ccc(cc2)CC(C(=O)Nc3ccccc3)C(=O)Nc4ccccc4 (Siliphaivanh et al.,2007)	0.047	1.328	0.600	b
**40**	Nc1ccccc1NC(=O)c2ccc(cc2)CC(C)(C(=O)Nc3ccccc3)C(=O)Nc4ccccc4 (Siliphaivanh et al.,2007)	0.200	0.699	0.430	b
**41**	Nc1ccccc1NC(=O)c2ccc(cc2)CNC(=O)c(c3)c(=O)oc(c34)cc(cc4)OCCC (Abdizadeh et al., 2017)	0.87	0.060	0.270	b
**42**	Nc1ccccc1NC(=O)c2ccc(cc2)CNC(=O)c(c3)c(=O)oc(c34)cc(cc4)OCc5ccc(Br)cc5 (Abdizadeh et al., 2017)	0.50	0.301	0.330	a
**43**	Nc1ccccc1NC(=O)c2ccc(cc2)CNC(=O)c(c3)c(=O)oc(c34)cc(cc4)OCc5ccc(cc5)OC (Abdizadeh et al., 2017)	0.71	0.149	0.100	a
**44**	Nc1ccccc1NC(=O)c2ccc(cc2)CNC(=O)c(c3)c(=O)oc(c34)cc(cc4)OCc5ccc(Cl)c(Cl)c5 (Abdizadeh et al., 2017)	0.47	0.328	0.330	a
**45**	Nc1ccccc1NC(=O)c2ccc(nc2)N(C)CCN(C)C(=O)OCc3ccccc3 (Hamblett et al., 2007)	0.963	0.016	0.070	a
**46**	Nc1ccccc1NC(=O)c2ccc(nc2)N3CCN(CC3)C(=O)OCc4ccccc4 (Hamblett et al., 2007)	0.168	0.775	1.010	a
**47**	Nc1ccccc1NC(=O)c2ccc(nc2)N3CCN[C@H](C3)C (Hamblett et al., 2007)	0.435	0.362	0.520	a
**48**	Nc1ccccc1NC(=O)c2ccc(nc2)N3CCN([C@H](C3)C)C(=O)OC(C)(C)C (Hamblett et al., 2007)	0.242	0.616	0.930	a
**49**	Nc1ccccc1NC(=O)c2ccc(nc2)N3CCN([C@H](C3)C)C(=O)OCc4ccccc4 (Hamblett et al., 2007)	0.073	1.137	1.100	b
**50**	Nc1ccccc1NC(=O)c2ccc(nc2)N3CCN([C@@H](C3)C)C(=O)OCc4ccccc4 (Hamblett et al., 2007)	0.040	1.398	1.140	a
**51**	Nc1ccccc1NC(=O)c2ccc(nc2)N(C3)CCN(C3(C)C)C(=O)OCc4ccccc4 (Hamblett et al., 2007)	0.169	0.772	0.860	a
**52**	Nc1ccccc1NC(=O)c2ccc(nc2)N3[C@H](C)CN([C@H](C3)C)C(=O)OCc4ccccc4 (Hamblettet al., 2007)	0.255	0.593	0.510	a

**53**	Nc1ccccc1NC(=O)c2ccc(nc2)N3CCN([C@H](C3)C(C)C)C(=O)OCc4ccccc4 (Hamblett et al., 2007)	0.326	0.487	0.740	a
**54**	Nc1ccccc1NC(=O)c2ccc(nc2)N(C3)CCN(C3(C)C)C(=O)OCCc4ccccc4 (Hamblett et al., 2007)	0.077	1.114	0.830	a
**55**	Nc1ccccc1NC(=O)c2ccc(nc2)N(C3)CCN(C3(C)C)S(=O)(=O)c4ccccc4 (Hamblett et al., 2007)	0.149	0.827	0.950	b
**56**	Nc1ccccc1NC(=O)c2ccc(nc2)N3CCN([C@H](C3)C)C(=O)Nc4ccccc4 (Hamblett et al., 2007)	0.075	1.125	0.910	b
**57**	Nc1ccccc1NC(=O)c2ccc(nc2)N3CCN([C@H](C3)C)C(=O)NCc4ccccc4 (Hamblett et al., 2007)	0.039	1.409	1.300	a
**58**	Nc1ccccc1NC(=O)c2ccc(nc2)N3CCN([C@H](C3)C)C(=O)NCc4ccc(cc4)OC (Hamblett et al., 2007)	0.047	1.328	1.360	a
**59**	Nc1ccccc1NC(=O)c2ccc(nc2)N3CCN([C@H](C3)C)C(=O)N[C@H](C)c4cc ccc4 (Hamblett et al., 2007)	0.101	0.996	1.280	a
**60**	Nc1ccccc1NC(=O)c2ccc(nc2)N3CCN([C@H](C3)C)C(=O)N[C@@H](C)c4 ccccc4 (Hamblett et al., 2007)	0.047	1.328	1.270	b
**61**	Nc1ccccc1NC(=O)c2ccc(nc2)N3CCN([C@H](C3)C)C(=O)NCCc4ccccc4 (Hamblett et al., 2007)	0.047	1.328	1.240	a
**62**	Nc1ccccc1NC(=O)c2ccc(nc2)N(CC3)CC(N34)CCCC4 (Hamblett et al., 2007)	0.588	0.231	0.250	a
**63**	Nc1ccccc1NC(=O)c2ccc(nc2)N(CC3)Cc(n34)ncc4-c5ccccc5 (Hamblett et al., 2007)	0.139	0.857	0.580	a
**64**	Nc1ccccc1NC(=O)c2ccc(nc2)N(CC3)Cc(n34)ncc4Cc5ccccc5 (Hamblett et al., 2007)	0.222	0.654	0.460	b
**65**	Nc1ccccc1NC(=O)c2ccc(nc2)N(C(C34)CC4)CCN3C(=O)OCc5ccccc5 (Hamblettet al., 2007)	0.140	0.854	1.050	b
**66**	Nc1ccccc1NC(=O)c2ccc(nc2)N(c(c34)cccc4)CCN3C(=O)OCc5ccccc5 (Hamblett et al., 2007)	0.092	1.036	1.080	b
**67**	Nc1ccccc1NC(=O)c2ccc(nc2)N3CCN[C@H](C3)CCC (Hamblett et al., 2007) (Ref9)	0.304	0.517	0.600	b
**68**	Nc1ccccc1NC(=O)c2ccc(nc2)N3CCN[C@H](C3)CCCC (Hamblett et al., 2007) (Ref9)	0.301	0.521	0.440	a
**69**	Nc1ccccc1NC(=O)c2ccc(nc2)N3[C@H](C)CN[C@H](C3)C (Hamblett et al., 2007)	0.582	0.235	0.300	a
**70**	Nc1ccccc1NC(=O)c2ccc(nc2)N(C3)CCNC3(C)C (Hamblett et al., 2007)	0.529	0.277	0.600	a
**71**	Nc1ccccc1NC(=O)c2ccc(nc2)N3CC(C)NC(C3)C (Hamblett et al., 2007)	0.821	0.086	0.400	a
**72**	Nc1ccccc1NC(=O)c2ccc(nc2)N3CCN(C(=O)OC(C)(C)C)C(C3)Cc4ccccc4	0.352	0.453	0.600	a

	(Hamblett et al., 2007)				
**73**	Nc1ccccc1NC(=O)c2ccc(nc2)N(CC34)C(C4)CN3<br>(Hamblett et al., 2007)	0.307	0.513	0.390	b
**74**	Nc1ccccc1NC(=O)c2ccc(nc2)N(CC34)C(C4)CN3C(=O)OC(C)(C)C<br>(Hamblett et al., 2007)	0.189	0.724	0.940	b
**75**	Nc1ccccc1NC(=O)c2ccc(nc2)N(CC34)C(C4)CN3C(=O)OCc5ccccc5<br>(Hamblett et al., 2007)	0.068	1.167	1.250	a
**76**	Nc1ccccc1NC(=O)c2ccc(nc2)N(CC34)C(C4)CN3Cc5ccccc5<br>(Hamblett et al., 2007)	0.235	0.629	0.390	b
**77**	Nc1ccccc1NC(=O)c2ccc(nc2)N(CC34)C(C4)CN3C(=O)CCc5ccccc5<br>(Hamblett et al., 2007)	0.167	0.777	0.750	a
**78**	Nc1ccccc1NC(=O)c2ccc(nc2)N(CC34)C(CN3)CC4<br>(Hamblett et al., 2007)	0.405	0.393	0.590	a
**79**	Nc1ccccc1NC(=O)c2ccc(nc2)N(CC34)C(CC4)CN3C(=O)OC(C)(C)C<br>(Hamblett et al., 2007)	0.669	0.175	0.480	a
**80**	Nc1ccccc1NC(=O)c2ccc(nc2)N(CC34)C(CC4)CN3C(=O)OCc5ccccc5<br>(Hamblett et al., 2007)	0.067	1.174	0.860	b
**81**	Nc1ccccc1NC(=O)c2ccc(nc2)N(CC34)C(CC4)CN3C(=O)OCc5cccnc5<br>(Hamblett et al., 2007)	0.091	1.041	0.930	a
**82**	Nc1ccccc1NC(=O)c2ccc(nc2)N(CC34)CC(CC4)N3C(=O)OC(C)(C)C<br>(Hamblett et al., 2007)	0.167	0.777	0.990	b
**83**	Nc1ccccc1NC(=O)c2ccc(nc2)N(CC34)CC(CC4)N3C(=O)OCc5ccccc5<br>(Hamblett et al., 2007)	0.082	1.086	1.110	a
**84**	Nc1ccccc1NC(=O)c2ncccc2<br>(Andrews and Stokes, et al., 2008)	2.455	-0.390	-0.310	a
**85**	Nc1ccccc1NC(=O)c2ccc(cc2)-c3sccn3<br>(Andrews and Stokes, et al., 2008)	0.123	0.910	0.650	a
**86**	Nc1ccccc1NC(=O)c2ccc(cc2)C3CCN(CC3)Cc4ccccc4<br>(Andrews and Stokes, et al., 2008)	0.045	1.347	1.120	a
**87**	Nc1ccccc1NC(=O)c2ccc(cc2)C3CCN(C)CC3<br>(Andrews and Stokes, et al., 2008)	0.138	0.860	0.990	b
**88**	Nc1ccccc1NC(=O)c2ccc(cc2)-c3ncc(s3)CN4CCCCC4<br>(Andrews and Stokes, et al., 2008)	0.022	1.658	1.570	a*
**89**	Nc1ccccc1NC(=O)c2ccc(cc2)-c3ncc(s3)CNCC<br>(Andrews and Stokes, et al., 2008)	0.038	1.420	1.450	b
**90**	Nc1ccccc1NC(=O)c2ccc(cc2)-c3ncc(s3)CNCCC<br>(Andrews and Stokes, et al., 2008)	0.028	1.553	1.420	a*
**91**	Nc1ccccc1NC(=O)c2ccc(cc2)C3CCN(CC3)CCC(=O)Nc4c(F)cccc4<br>(Andrews and Stokes, et al., 2008)	0.068	1.167	1.050	a
**92**	Nc1ccccc1NC(=O)c2ccc(cc2)C3CCN(CC3)Cc4ccc(cc4)C(=O)N(C)C<br>(Andrews and Stokes, et al., 2008)	0.085	1.071	1.140	a

**93**	Nc1ccccc1NC(=O)c2ccc(cc2)C3CCN(CC3)Cc4ccc(cc4)C(=O)NC5CC5 (Andrews and Stokes, et al., 2008)	0.058	1.237	1.180	a
**94**	Nc1ccccc1NC(=O)c2ccc(cc2)C3CCN(CC3)Cc4ccc(cc4)C(=O)NCC (Andrews and Stokes, et al., 2008)	0.035	1.456	1.130	b*
**95**	Nc1ccccc1NC(=O)c2ccc(cc2)C3CCN(CC3)Cc4ccc(cc4)C(=O)NC (Andrews and Stokes, et al., 2008)	0.031	1.509	1.150	b*
**96**	Nc1ccccc1NC(=O)c2ccc(cc2)-c3c(C#N)cc(cn3)N (Andrews and Gibson, et al., 2008)	0.012	1.921	1.670	a*
**97**	C1CCN1Cc(cn2)cc(C)c2-c3ccc(cc3)C(=O)Nc4c(N)cccc4 (Andrews and Gibson, et al., 2008)	0.021	1.678	1.660	a*
**98**	C1CCN1Cc(cn2)cc(Cl)c2-c3ccc(cc3)C(=O)Nc4c(N)cccc4 (Andrews and Gibson, et al., 2008)	0.019	1.721	1.620	a*
**99**	C1CCN1Cc(cn2)cc(F)c2-c3ccc(cc3)C(=O)Nc4c(N)cccc4 (Andrews and Gibson, et al., 2008)	0.05	1.301	1.410	a
**100**	c1cccc(N)c1NC(=O)c(cc2)ccc2-c3c(C)cc(cn3)CN4CCN(CC4)CC (Andrews and Gibson, et al., 2008)	0.019	1.721	1.710	a*
**101**	c1cccc(N)c1NC(=O)c(cc2)ccc2-c3c(Cl)cc(cn3)CN4CCN(CC4)CC (Andrews and Gibson, et al., 2008)	0.016	1.796	1.730	a*
**102**	c1cccc(N)c1NC(=O)c(cc2)ccc2-c3c(F)cc(cn3)CN4CCN(CC4)CC (Andrews and Gibson, et al., 2008)	0.038	1.420	1.330	a
**103**	c1cccc(N)c1NC(=O)c(cc2)ccc2-c3c(C)cc(cn3)CN4CCN(CC4)C(C)C (Andrews and Gibson, et al., 2008)	0.019	1.721	1.790	a*
**104**	c1cccc(N)c1NC(=O)c(cc2)ccc2-c3c(Cl)cc(cn3)CN4CCN(CC4)C(C)C (Andrews and Gibson, et al., 2008)	0.013	1.886	1.700	a*
**105**	c1cccc(N)c1NC(=O)c(cc2)ccc2-c3c(F)cc(cn3)CN4CCN(CC4)C(C)C (Andrews and Gibson, et al., 2008)	0.033	1.481	1.250	b*
**106**	s1cccc1-c(ccc2N)cc2NC(=O)c3ccc(cc3)CN(CCO)Cc(c4)ccc(c45)OCO5 (Hirata, et al., 2012)	0.06	1.222	1.410	a
**107**	Nc1ccccc1NC(=O)c2ccc(cc2)CN(CCO)Cc(c3)ccc(c34)OCO4 (Hirata, et al., 2012)	1.17	-0.068	0.250	a
**108**	s1cccc1-c(ccc2N)cc2NC(=O)c3ccc(cc3)CNC(=O)CC (Hirata, et al., 2012)	0.04	1.398	1.500	a
**109**	s1cccc1-c(ccc2N)cc2NC(=O)c3ccc(cc3)CNC(=O)N4CCOCC4 (Hirata, et al., 2012)	0.10	1.000	1.120	a
**110**	s1cccc1-c(ccc2N)cc2NC(=O)c3ccc(cc3)CNC(=O)N4CCN(C(=O)C4=O)CC (Hirata, et al., 2012)	0.05	1.301	0.960	a
**111**	s1cccc1-c(ccc2N)cc2NC(=O)c3ccc(cc3)CNC(=O)N4CCN(C)CC4 (Hirata, et al., 2012)	0.08	1.097	1.140	a
**112**	c1ccccc1-c(ccc2O)cc2NC(=O)c3ccccc3 (Methot and Chakravarty, et al., 2008)	0.058	1.237	1.090	b
**113**	c1ccccc1-c(ccc2O)cc2NC(=O)c3ccccn3	0.31	0.509	0.760	a

	(Methot and Chakravarty, et al., 2008)				
**114**	c1ccccc1-c(ccc2O)cc2NC(=O)c3cccnc3 (Methot and Chakravarty, et al., 2008)	0.068	1.167	0.980	b
**115**	c1ccccc1-c(ccc2O)cc2NC(=O)c3ccncc3 (Methot and Chakravarty, et al., 2008)	0.075	1.125	0.960	a
**116**	c1ccccc1-c(ccc2O)cc2NC(=O)c3sccc3 (Methot and Chakravarty, et al., 2008)	0.13	0.886	1.070	b
**117**	c1ccccc1-c(ccc2O)cc2NC(=O)c(c3)sc(c34)cccc4 (Methot and Chakravarty, et al., 2008)	0.13	0.886	1.240	a
**118**	c1ccccc1-c(ccc2O)cc2NC(=O)c3ccco3 (Methot and Chakravarty, et al., 2008)	0.13	0.886	0.860	b
**119**	c1ccccc1-c(ccc2O)cc2NC(=O)c3ccno3 (Methot and Chakravarty, et al., 2008)	0.10	1.000	0.800	a
**120**	c1ccccc1-c(ccc2O)cc2NC(=O)CCCc3ccccc3 (Methot and Chakravarty, et al., 2008)	0.61	0.215	0.110	a
**121**	c1ccccc1-c(ccc2O)cc2NC(=O)C/C=C/c3ccccc3 (Methot and Chakravarty, et al., 2008)	1.2	-0.079	0.230	a
**122**	c1cocc1-c(ccc2N)cc2NC(=O)c3cccnc3 (Methot and Chakravarty, et al., 2008)	0.16	0.796	0.430	b
**123**	c1cncn1-c(ccc2N)cc2NC(=O)c3cccnc3 (Methot and Chakravarty, et al., 2008)	1.00	0.000	0.090	a
**124**	c1cnccc1-c(ccc2N)cc2NC(=O)c3cccnc3 (Methot and Chakravarty, et al., 2008)	0.65	0.187	0.360	a
**125**	C1CCCN1c(ccc2N)cc2NC(=O)c3cccnc3 (Methot and Chakravarty, et al., 2008)	0.67	0.174	0.500	a
**126**	c1cc(F)ccc1-c(ccc2N)cc2NC(=O)c3cccnc3 (Methot and Chakravarty, et al., 2008)	0.14	0.854	0.460	a
**127**	c1cc(N)ccc1-c(ccc2N)cc2NC(=O)c3cccnc3 (Methot and Chakravarty, et al., 2008)	0.33	0.481	0.150	a
**128**	c1cc(N(C)C)ccc1-c(ccc2N)cc2NC(=O)c3cccnc3 (Methot and Chakravarty, et al., 2008)	5.3	-0.724	-0.650	a
**129**	N#CCc(cc1)ccc1-c(ccc2N)cc2NC(=O)c3cccnc3 (Methot and Chakravarty, et al., 2008)	0.25	0.602	0.210	a
**130**	c1cc(CN)ccc1-c(ccc2N)cc2NC(=O)c3cccnc3 (Methot and Chakravarty, et al., 2008)	2.1	-0.322	-0.020	a
**131**	c1ncccc1C(=O)Nc2cc(ccc2N)-c3ccc(cc3)CCC(=O)OC (Methot and Chakravarty, et al., 2008)	1.5	-0.176	0.160	b
**132**	c1ncccc1C(=O)Nc2cc(ccc2N)-c3ccc(cc3)N4CCN(CC4)c5ccccc5 (Methot and Chakravarty, et al., 2008)	50	-1.699	-2.070	a
**133**	Nc1ccccc1NC(=O)c2ccccc2 (Methot and Chakravarty, et al., 2008)	2.4	-0.380	-0.030	a

**134**	c1ccccc1-c(ccc2N)cc2NC(=O)c3ccccc3<br>(Methot and Chakravarty, et al., 2008)	0.060	1.222	0.940	a
**135**	c1ccsc1-c(ccc2N)cc2NC(=O)c3ccccc3<br>(Methot and Chakravarty, et al., 2008)	0.048	1.319	0.900	b
**136**	c1ccccc1-<br>c(ccc2N)cc2NC(=O)c3ccc(nc3)N4CCN([C@H](C4)C)C(=O)OCc5ccccc5<br>(Methot and Chakravarty, et al., 2008)	0.059	1.229	1.410	a
**137**	Nc1ccccc1NC(=O)c2ccc(cc2)NC(=O)C<br>**(CI-994)**<br>(Methot and Chakravarty, et al., 2008)	0.57	0.244	0.790	a
**138**	c1ccccc1-c(ccc2O)cc2NC(=O)c3ccc(cc3)NC(=O)C<br>(Methot and Chakravarty, et al., 2008)	0.018	1.745	1.850	a*
**139**	c1ccccc1-c(ccc2N)cc2NC(=O)c3ccc(cc3)NC(=O)C<br>(Methot and Chakravarty, et al., 2008)	0.028	1.553	1.800	b*
**140**	Nc1ccccc1NC(=O)c2ccc(cc2)CNCc3ccc(cc3)-c4cccs4<br>(Kiyokawa, et al., 2010)	1.00	0.000	0.070	a
**141**	Nc1ccccc1NC(=O)c2ccc(cc2)CNCc(s3)cc(c34)cccc4<br>(Kiyokawa, et al., 2010)	1.1	-0.041	0.020	a
**142**	Nc1ccccc1NC(=O)c2ccc(cc2)CNCc3cc(ccc3)-c4cccs4<br>(Kiyokawa, et al., 2010)	3.3	-0.519	-0.460	a
**143**	Nc1ccccc1NC(=O)c2ccc(cc2)CNCc3ccc(cc3)-c4ccccc4<br>(Kiyokawa, et al., 2010)	0.9	0.046	0.170	b
**144**	Nc1ccccc1NC(=O)c2ccc(cc2)CN(C)Cc3ccc(cc3)-c4cccs4<br>(Kiyokawa, et al., 2010)	10	-1.000	-0.810	a
**145**	Nc1ccccc1NC(=O)c2ccc(cc2)CN(S(=O)(=O)C)Cc3ccc(cc3)-c4cccs4<br>(Kiyokawa, et al., 2010)	1.2	-0.079	0.070	a
**146**	Nc1ccccc1NC(=O)c2ccc(cc2)CN(C(=O)N(C)C)Cc3ccc(cc3)-c4cccs4<br>(Kiyokawa, et al., 2010)	0.8	0.097	0.130	a
**147**	Nc1ccccc1NC(=O)c2ccc(cc2)CN(CCO)Cc3ccc(cc3)-c4cccs4<br>(Kiyokawa, et al., 2010)	0.8	0.097	0.010	b
**148**	Nc1ccccc1NC(=O)c2ccc(cc2)CN(CCO)Cc3ccc(cc3)-c4ccccc4<br>(Kiyokawa, et al., 2010)	0.7	0.155	0.860	b
**149**	c1ccccc1-c(c2)ccc(O)c2NC(=O)c3ccc(cc3)CNCC(C)C<br>(Kattar, et al., 2009)	0.112	0.951	1.250	b
**150**	c1ccccc1-c(c2)ccc(O)c2NC(=O)c3ccc(cc3)CNCc4cnccc4<br>(Kattar, et al., 2009)	0.032	1.495	1.740	b*
**151**	c1ccccc1-c(c2)ccc(O)c2NC(=O)c3ccc(cc3)CNC(C)c4ccncc4<br>(Kattar, et al., 2009)	0.013	1.886	2.080	a*
**152**	c1c(Cl)ccc(O)c1NC(=O)c2ccc(cc2)CNc3ccncc3<br>(Kattar, et al., 2009)	5.032	-0.702	-0.740	a
**153**	c1c(F)ccc(O)c1NC(=O)c2ccc(cc2)CNc3ccncc3	2.013	-0.304	-0.320	a

	(Kattar, et al., 2009)				
**154**	c1c(Br)ccc(O)c1NC(=O)c2ccc(cc2)CNc3ccncc3<br>(Kattar, et al., 2009)	5.223	-0.718	-0.700	a
**155**	c1cccc(O)c1NC(=O)c2ccc(cc2)CNc3ccncc3<br>(Kattar, et al., 2009)	1.568	-0.195	-0.350	a
**156**	COc(c1)ccc(O)c1NC(=O)c2ccc(cc2)CNc3ccncc3<br>(Kattar, et al., 2009)	7.964	-0.901	-0.490	a
**157**	c1ccccc1-c(c2)ccc(N)c2NC(=O)c3ccc(cc3)CNCc4c(C)n(C)nc4<br>(Kattar, et al., 2009	0.013	1.886	1.860	a*
**158**	c1ccccc1-c(c2)ccc(N)c2NC(=O)c3ccc(cc3)CN(CC4)CCC45CNCC5<br>(Kattar, et al., 2009)	0.033	1.481	1.650	a*
**159**	c1cccc(F)c1-c(c2)ccc(N)c2NC(=O)c3ccc(cc3)CN(CC4)CCC45NCCC5<br>(Kattar, et al., 2009)	0.016	1.796	2.110	a*
**160**	c1ccc(F)cc1-c(c2)ccc(N)c2NC(=O)c3ccc(cc3)CN(CC4)CCC45NCCC5<br>(Kattar, et al., 2009)	0.047	1.328	2.180	b
**161**	c1cc(F)ccc1-c(c2)ccc(N)c2NC(=O)c3ccc(cc3)CN(CC4)CCC45NCCC5<br>(Kattar, et al., 2009)	0.013	1.886	1.730	a*
**162**	s1cccc1-c(c2)ccc(N)c2NC(=O)c3ccc(cc3)C(=O)N(CC4)CCC45NCCC5<br>(Kattar, et al., 2009)	0.011	1.959	1.980	b*
**163**	c1cccc(F)c1-c(c2)ccc(N)c2NC(=O)c3ccc(cc3)C(=O)N(CC4)CCC45NCCC5<br>(Kattar, et al., 2009)	0.011	1.959	2.040	a*
**164**	c1cc(F)ccc1-c(c2)ccc(N)c2NC(=O)c3ccc(cc3)C(=O)N(CC4)CCC45NCCC5<br>(Kattar, et al., 2009)	0.012	1.921	1.640	a*
**165**	c1ncccc1COC(=O)NCc2ccc(cc2)C(=O)Nc3c(N)cccc3<br>(Mahboobi, et al., 2009)	0.74	0.131	0.290	a
**166**	c1ncccc1-c(cn2)cnc2Nc3cc(ccc3C)NC(=O)c4ccc(s4)C(=O)Nc5c(N)cccc5<br>(Mahboobi, et al., 2009)	0.23	0.638	0.720	a
**167**	c1ncccc1-c2nc(sc2)Nc3cc(ccc3C)NC(=O)c4cccc(c4)C(=O)Nc5c(N)cccc5<br>(Mahboobi, et al., 2009)	0.17	0.770	0.890	a
**168**	c1ncccc1-c2nc(sc2)Nc3cc(ccc3)NC(=O)c4cccc(n4)C(=O)Nc5c(N)cccc5<br>(Mahboobi, et al., 2009)	0.45	0.347	0.360	a
**169**	c1ncccc1-c2nc(sc2)Nc3cc(ccc3C)NC(=O)c4cccc(n4)C(=O)Nc5c(N)cccc5<br>(Mahboobi, et al., 2009)	0.31	0.509	0.290	b
**170**	c1ncccc1-c2nc(sc2)Nc3cc(ccc3C)NC(=O)c4ccc(s4)C(=O)Nc5c(N)cccc5<br>(Mahboobi, et al., 2009)	1.13	-0.053	0.200	a
**171**	c1ccccc1N(CNC2=O)C23CCN(CC3)c(nc4)ccc4C(=O)Nc5ccccc5N<br>(Methot and Hamblett, et al., 2008)	0.039	1.409	1.520	a
**172**	c1ccccc1S(=O)(=O)N(C2)CCC23CN(CC3)c(nc4)ccc4C(=O)Nc5ccccc5N<br>(Methot and Hamblett, et al., 2008)	0.120	0.921	0.940	a
**173**	c1cccc(NC2)c1C23CCN(CC3)c(nc4)ccc4C(=O)Nc5ccccc5N<br>(Methot and Hamblett, et al., 2008)	0.130	0.886	0.940	b

**174**	c1cccc(CNC2)c1C23CCN(CC3)c(nc4)ccc4C(=O)Nc5ccccc5N (Methot and Hamblett, et al., 2008)	0.089	1.051	1.080	a
**175**	s1cccc1-c(ccc2N)cc2NC(=O)c3cccnc3 (Methot and Hamblett, et al., 2008)	0.065	1.187	0.580	a
**176**	C1CNCC12CCN(CC2)c(nc3)ccc3C(=O)Nc4ccccc4N (Methot and Hamblett, et al., 2008)	0.160	0.796	0.630	b
**177**	C1CNCC12CCN(CC2)c(nc3)ccc3C(=O)Nc4cc(ccc4N)-c5ccccc5 (Methot and Hamblett, et al., 2008)	0.011	1.959	1.890	b*
**178**	C1CNCC12CCN(CC2)c(nc3)ccc3C(=O)Nc4cc(ccc4N)-c5cn(C)nc5 (Methot and Hamblett, et al., 2008)	0.036	1.444	1.480	a
**179**	C1CNCC12CCN(CC2)c(nc3)ccc3C(=O)Nc4cc(ccc4N)-c5cc[nH]n5 (Methot and Hamblett, et al., 2008)	0.018	1.745	1.590	a*
**180**	C1CNCC12CCN(CC2)c(nc3)ccc3C(=O)Nc4cc(ccc4N)-c5cn(cn5)C (Methot and Hamblett, et al., 2008)	0.022	1.658	1.690	a*
**181**	CCNC(=O)N(CC1)CC12CCN(CC2)c(nc3)ccc3C(=O)Nc4cc(ccc4N)-c5cccs5 (Methot and Hamblett, et al., 2008)	0.014	1.854	1.830	a*
**182**	n1cccnc1N(CC2)CC23CCN(CC3)c(nc4)ccc4C(=O)Nc5cc(ccc5N)-c6cccs6 (Methot and Hamblett, et al., 2008)	0.028	1.553	1.590	a*
**183**	FC(F)(F)CN(CC1)CC12CCN(CC2)c(nc3)ccc3C(=O)Nc4cc(ccc4N)-c5cccs5 (Methot and Hamblett, et al., 2008)	0.017	1.770	1.980	b*
**184**	OC(=O)CN(CC1)CC12CCN(CC2)c(nc3)ccc3C(=O)Nc4cc(ccc4N)-c5cccs5 (Methot and Hamblett, et al., 2008)	0.017	1.770	1.770	a*
**185**	NC(=O)CN(CC1)CC12CCN(CC2)c(nc3)ccc3C(=O)Nc4cc(ccc4N)-c5cccs5 (Methot and Hamblett, et al., 2008)	0.013	1.886	1.790	b*
**186**	CC(=O)N(C1)CCC12CN(CC2)c(nc3)ccc3C(=O)Nc4cc(ccc4N)-c5cccs5 (Methot and Hamblett, et al., 2008)	0.012	1.921	1.950	a*
**187**	CC(=O)N(C1)CC12CCN(CC2)c(nc3)ccc3C(=O)Nc4cc(ccc4N)-c5cccs5 (Methot and Hamblett, et al., 2008)	0.023	1.638	1.910	b*
**188**	C1CCN(C(=O)C)C12CCN(CC2)c(nc3)ccc3C(=O)Nc4cc(ccc4N)-c5cccs5 (Methot and Hamblett, et al., 2008)	0.023	1.638	1.940	a*
**189**	C1CCC(=O)C12CCN(CC2)c(nc3)ccc3C(=O)Nc4cc(ccc4N)-c5cccs5 (Methot and Hamblett, et al., 2008)	0.013	1.886	1.740	a*
**190**	C1CCC(O)C12CCN(CC2)c(nc3)ccc3C(=O)Nc4cc(ccc4N)-c5cccs5 (Methot and Hamblett, et al., 2008)	0.029	1.538	2.020	b*
**191**	C1CNC(=O)C12CCN(CC2)c(nc3)ccc3C(=O)Nc4cc(ccc4N)-c5cccs5 (Methot and Hamblett, et al., 2008)	0.021	1.678	1.950	a*
**192**	C1NC(=O)C12CCN(CC2)c(nc3)ccc3C(=O)Nc4cc(ccc4N)-c5cccs5 (Methot and Hamblett, et al., 2008)	0.011	1.959	2.110	b*
**193**	C1C(=O)NCC12CCN(CC2)c(nc3)ccc3C(=O)Nc4cc(ccc4N)-c5cccs5 (Methot and Hamblett, et al., 2008)	0.013	1.886	2.000	a*
**194**	N1CNC(=O)C12CCN(CC2)c(nc3)ccc3C(=O)Nc4cc(ccc4N)-c5cccs5	0.011	1.959	2.280	b*

	(Methot and Hamblett, et al., 2008				
**195**	c1ccccc1NC(=O)CNCCCC(=O)Nc2ccccc2N (Rajak, et al., 2012)	9.2	-0.964	-0.890	a
**196**	Brc1ccc(cc1)NC(=O)CNCCCC(=O)Nc2ccccc2N (Rajak, et al., 2012)	1.7	-0.230	-0.160	a
**197**	Clc1ccccc1NC(=O)CNCCCC(=O)Nc2ccccc2N (Rajak, et al., 2012)	2.9	-0.462	-0.470	a
**198**	Clc1cccc(c1)NC(=O)CNCCCC(=O)Nc2ccccc2N (Rajak, et al., 2012)	1.8	-0.255	-0.060	a
**199**	Clc1ccc(cc1)NC(=O)CNCCCC(=O)Nc2ccccc2N (Rajak, et al., 2012)	1.4	-0.146	-0.140	b
**200**	O=[N+]([O-])c1ccccc1NC(=O)CNCCCC(=O)Nc2ccccc2N (Rajak, et al., 2012)	3.1	-0.491	-0.360	a
**201**	O=[N+]([O-])c1cccc(c1)NC(=O)CNCCCC(=O)Nc2ccccc2N (Rajak, et al., 2012)	2.3	-0.362	-0.120	b
**202**	O=[N+]([O-])c1ccc(cc1)NC(=O)CNCCCC(=O)Nc2ccccc2N (Rajak, et al., 2012)	0.9	0.046	-0.230	a
**203**	COc1ccc(cc1)NC(=O)CNCCCC(=O)Nc2ccccc2N (Rajak, et al., 2012)	3.6	-0.556	-0.710	b
**204**	c1ccccc1NC(=O)CNc2ccc(cc2)C(=O)Nc3c(N)cccc3 (Rajak, et al., 2012)	7.4	-0.869	-0.670	a
**205**	Brc1ccc(cc1)NC(=O)CNc2ccc(cc2)C(=O)Nc3c(N)cccc3 (Rajak, et al., 2012)	1.6	-0.204	-0.500	b
**206**	Clc1ccccc1NC(=O)CNc2ccc(cc2)C(=O)Nc3c(N)cccc3 (Rajak, et al., 2012)	2.9	-0.462	-0.400	a
**207**	Clc1cccc(c1)NC(=O)CNc2ccc(cc2)C(=O)Nc3c(N)cccc3 (Rajak, et al., 2012)	2.5	-0.398	-0.590	a
**208**	O=[N+]([O-])c1ccccc1NC(=O)CNc2ccc(cc2)C(=O)Nc3c(N)cccc3 (Rajak, et al., 2012)	2.00	-0.301	-0.550	a
**209**	COc1ccc(cc1)NC(=O)CNc2ccc(cc2)C(=O)Nc3c(N)cccc3 (Rajak, et al., 2012)	3.1	-0.491	-0.490	b
**210**	Cc1ccc(cc1)NC(=O)CNc2ccc(cc2)C(=O)Nc3c(N)cccc3 (Rajak, et al., 2012)	4.5	-0.653	-0.480	b
**211**	Clc1ccc(Cl)c(c1)NC(=O)CNc2ccc(cc2)C(=O)Nc3c(N)cccc3 (Rajak, et al., 2012)	8.6	-0.934	-0.710	a
**212**	c1cccc(N)c1NC(=O)\C=C\c(cc2)ccc2-c3nnc(o3)Cc4cccc(c45)cccc5 (Valente, et al., 2014)	1.00	0.000	-0.220	a
**213**	c1cccc(N)c1NC(=O)\C=C\c(cc2)ccc2-c3nnc(o3)Cc(c4)ccc(c45)cccc5 (Valente, et al., 2014)	2.4	-0.380	-0.570	a
**214**	c1cccc(N)c1NC(=O)\C=C\c(cc2)ccc2-c3nnc(o3)Cc4c[nH]c(c45)cccc5 (Valente, et al., 2014)	1.5	-0.176	-0.200	a

**215**	c1cccc(N)c1NC(=O)\C=C\c(cc2)ccc2Cc3nnc(o3)Cc4cccc(c45)cccc5 (Valente, et al., 2014)	4.1	-0.613	-0.620	a
**216**	c1cccc(N)c1NC(=O)c(cc2)ccc2-c3nnc(o3)Cc4cccc(c45)cccc5 (Valente, et al., 2014)	0.3	0.523	0.250	a
**217**	c1cccc(N)c1NC(=O)c(cc2)ccc2Cc3nnc(o3)Cc4cccc(c45)cccc5 (Valente, et al., 2014)	0.2	0.699	0.240	b
**218**	s1ccnc1C(=O)NCCCCCC(=O)Nc2ccc(F)cc2N (Rusche, et al., 2016)	18	-1.255	-1.480	a
**219**	Nc1ccccc1NC(=O)CCCCCNC(=O)C(=N2)C(C)c(c23)cccc3 (Rusche, et al., 2016)	0.371	0.431	0.720	a
**220**	Nc1ccccc1NC(=O)CCCCCNC(=O)C(=N2)Cc(c23)c(OC)ccc3 (Rusche, et al., 2016)	0.299	0.524	0.570	a
**221**	Nc1ccccc1NC(=O)CCCCCNC(=O)C(=N2)Cc(c23)c(ccc3)OCC (Rusche, et al., 2016)	0.262	0.582	0.590	a
**222**	Nc1ccccc1NC(=O)CCCCCNC(=O)C(=N2)Cc(c23)c(F)ccc3 (Rusche, et al., 2016)	0.436	0.361	0.540	a
**223**	Nc1ccccc1NC(=O)CCCCCNC(=O)C(=N2)Cc(c23)c(Cl)ccc3 (Rusche, et al., 2016)	0.254	0.595	0.530	a
**224**	Nc1ccccc1NC(=O)CCCCCNC(=O)C(=N2)Cc(c23)cc(cc3)OC(F)(F)F (Rusche, et al., 2016)	0.550	0.260	0.380	a
**225**	Nc1ccccc1NC(=O)CCCCCNC(=O)C(=N2)Cc(c23)cc(C)cc3 (Rusche, et al., 2016)	0.426	0.371	0.470	a
**226**	Nc1ccccc1NC(=O)CCCCCNC(=O)C(=N2)Cc(c23)cc(F)cc3 (Rusche, et al., 2016)	0.384	0.416	0.350	b
**227**	Nc1ccccc1NC(=O)CCCCCNC(=O)C(=N2)Cc(c23)cc(Cl)cc3 (Rusche, et al., 2016)	0.203	0.693	0.610	a
**228**	Nc1ccccc1NC(=O)CCCCCNC(=O)C(=N2)Cc(c23)cccc3OC (Rusche, et al., 2016)	0.392	0.407	0.490	b
**229**	Nc1ccccc1NC(=O)CCCCCNC(=O)C(=N2)Cc(c23)cccc3C (Rusche, et al., 2016)	0.059	1.229	0.640	b
**230**	Nc1ccccc1NC(=O)CCCCCNC(=O)C(=N2)Cc(c23)cccc3F (Rusche, et al., 2016)	0.079	1.102	1.180	a
**231**	Nc1ccccc1NC(=O)CCCCCNC(=O)C(=N2)Cc(c23)ccc(c3)N(C)C (Rusche, et al., 2016)	0.081	1.092	1.260	a
**232**	Nc1ccccc1NC(=O)CCCCCNC(=O)C(=N2)Cc(c23)c(OC(F)F)ccc3 (Rusche, et al., 2016)	0.041	1.387	1.500	a
**233**	Nc1ccccc1NC(=O)CCCCCNC(=O)c(n2C)cc(c23)cccc3 (Rusche, et al., 2016)	0.135	0.870	0.580	a
**234**	Nc1ccccc1NC(=O)CCCCCNC(=O)c(n2C)cc(c23)cc(F)cc3 (Rusche, et al., 2016)	0.081	1.092	0.810	a
**235**	Nc1ccccc1NC(=O)CCCCCNC(=O)c(n2C)cc(c23)cc(Cl)cc3	0.061	1.215	0.770	b

	(Rusche, et al., 2016)				
**236**	Nc1ccccc1NC(=O)CCCCCNC(=O)c(n2CCOC)cc(c23)cccc3<br>(Rusche, et al., 2016)	0.195	0.710	0.850	a
**237**	Nc1ccccc1NC(=O)CCCCCNC(=O)c2ccccc2CC<br>(Rusche, et al., 2016)	3.756	-0.575	-0.570	a
**238**	Nc1ccccc1NC(=O)CCCCCNC(=O)c2cccc(c2)N(C)C<br>(Rusche, et al., 2016)	0.585	0.233	0.270	b
**239**	Nc1ccccc1NC(=O)CCCCCNC(=O)C=2CN=c(c23)c(OC)ccc3<br>(Rusche, et al., 2016)	0.196	0.708	0.520	b
**240**	Nc1ccccc1NC(=O)CCCCCNC(=O)c(cc2)cc(c23)n(C)cc3<br>(Rusche, et al., 2016)	0.378	0.423	0.540	a
**241**	Nc1ccccc1NC(=O)CCCCCNC(=O)c(ccc2)c(c23)[nH]c(C)c3C<br>(Rusche, et al., 2016)	0.207	0.684	0.600	a
**242**	c1ccc(OC(F)(F)F)cc1C(=O)NCCCCCC(=O)Nc2c(N)cccc2<br>(Rusche, et al., 2016)	0.468	0.330	0.250	a
**243**	FC(F)(F)Oc(cc1)ccc1C(=O)NCCCCCC(=O)Nc2c(N)cccc2<br>(Rusche, et al., 2016)	0.501	0.300	0.180	a
**244**	c1cccc(c12)n(C)cc2C(=O)NCCCCCC(=O)Nc3c(N)cccc3<br>(Rusche, et al., 2016)	0.174	0.759	0.600	a
**245**	CCOc(cc1)cc(c12)CC(=N2)C(=O)NCCCCCC(=O)Nc3c(N)cccc3<br>(Rusche, et al., 2016)	0.359	0.445	0.590	a
**246**	CCNc(cc1)ccc1C(=O)NCCCCCC(=O)Nc2c(N)cccc2<br>(Rusche, et al., 2016)	0.403	0.395	0.660	a
**247**	Cc1c(C)[nH]c(c12)ccc(c2)C(=O)NCCCCCC(=O)Nc3c(N)cccc3<br>(Rusche, et al., 2016)	0.270	0.569	0.070	b
**248**	c1ccc(Cl)c(c12)=NCC=2C(=O)NCCCCCC(=O)Nc3c(N)cccc3<br>(Rusche, et al., 2016)	0.172	0.764	0.700	a
**249**	c1cc(C)ccc1C(=O)NCCCCCC(=O)Nc2c(O)cccc2<br>(Rusche, et al., 2016)	0.224	0.650	0.760	a
**250**	c1c(C)cccc1C(=O)NCCCCCC(=O)Nc2c(N)cccc2<br>(Rusche, et al., 2016)	0.527	0.278	0.250	b
**251**	Cc1cc(C)ccc1C(=O)NCCCCCC(=O)Nc2c(N)cccc2<br>(Rusche, et al., 2016)	2.143	-0.331	-0.210	a
**252**	c1cc(C(F)F)ccc1C(=O)NCCCCCC(=O)Nc2c(N)cccc2<br>(Rusche, et al., 2016)	0.362	0.441	0.180	a
**253**	c1cc(C(C)(C)O)ccc1C(=O)NCCCCCC(=O)Nc2c(N)cccc2<br>(Rusche, et al., 2016)	0.402	0.396	0.430	b
**254**	C1CCN1c(cc2)ccc2C(=O)NCCCCCC(=O)Nc3c(N)cccc3<br>(Rusche, et al., 2016)	0.272	0.565	0.530	a
**255**	C1CN(C)CCN1c(c2)cccc2C(=O)NCCCCCC(=O)Nc3c(N)cccc3<br>(Rusche, et al., 2016)	0.365	0.438	0.400	a

**256**	C1CN(C)CCN1c2ccccc2C(=O)NCCCCCC(=O)Nc3c(N)cccc3 (Rusche, et al., 2016)	3.581	-0.554	-0.650	a
**257**	C1COCCN1c(cc2)ccc2C(=O)NCCCCCC(=O)Nc3c(N)cccc3 (Rusche, et al., 2016)	0.334	0.476	0.560	a
**258**	c1cccc(Cl)c1C(=O)NCCCCCC(=O)Nc2c(N)cccc2 (Rusche, et al., 2016)	2.035	-0.309	-0.150	a
**259**	c1c(F)c(F)ccc1C(=O)NCCCCCC(=O)Nc2c(N)cccc2 (Rusche, et al., 2016)	0.772	0.112	-0.200	b
**260**	c1cccc(c12)=NCC=2C(=O)NCCCCCC(=O)Nc3c(N)cccc3 (Rusche, et al., 2016)	0.246	0.609	0.470	a
**261**	COc(c1)ccc(c12)=NCC=2C(=O)NCCCCCC(=O)Nc3c(N)cccc3 (Rusche, et al., 2016)	0.218	0.662	0.640	b
**262**	C1CCCCC1c2ccccc2C(=O)NCCCCCC(=O)Nc3c(N)cccc3 (Rusche, et al., 2016)	2.768	-0.442	-0.260	a
**263**	c1cccc(COC)c1C(=O)NCCCCCC(=O)Nc2c(N)cccc2 (Rusche, et al., 2016)	3.278	-0.516	-0.370	a
**264**	c1cc(OC)cc(c12)=NCC=2C(=O)NCCCCCC(=O)Nc3c(N)cccc3 (Rusche, et al., 2016)	0.248	0.606	0.450	b
**265**	N1=CCc(c12)c(ccc2)C(=O)NCCCCCC(=O)Nc3c(N)cccc3 (Rusche, et al., 2016)	0.524	0.281	0.150	b
**266**	Cn1ccc(c12)c(ccc2)C(=O)NCCCCCC(=O)Nc3c(N)cccc3 (Rusche, et al., 2016)	0.448	0.349	0.340	a
**267**	Nc1ccccc1NC(=O)CCCCCNC(=O)c(c2)ccc(c23)[nH]cc3 (Rusche, et al., 2016)	0.210	0.678	0.370	a
**268**	c1cn(C)c(c12)ccc(c2)C(=O)NCCCCCC(=O)Nc3c(N)cccc3 (Rusche, et al., 2016)	0.218	0.662	0.280	b
**269**	c1cccc(c12)n(C)nc2C(=O)NCCCCCC(=O)Nc3c(N)cccc3 (Rusche, et al., 2016)	0.299	0.524	0.530	a
**270**	c1n[nH]c(c12)c(ccc2)C(=O)NCCCCCC(=O)Nc3c(N)cccc3 (Rusche, et al., 2016)	0.371	0.431	0.480	a
**271**	C=CCc1ccccc1C(=O)NCCCCCC(=O)Nc2c(N)cccc2 (Rusche, et al., 2016)	2.698	-0.431	-0.440	a
**272**	CCOc1ccccc1C(=O)NCCCCCC(=O)Nc2c(N)cccc2 (Rusche, et al., 2016)	0.549	0.260	0.360	b
**273**	CCCOc1ccccc1C(=O)NCCCCCC(=O)Nc2c(N)cccc2 (Rusche, et al., 2016)	0.399	0.399	0.290	a
**274**	c1cccc(SCC)c1C(=O)NCCCCCC(=O)Nc2c(N)cccc2 (Rusche, et al., 2016)	1.333	-0.125	-0.140	a
**275**	O=S(=O)(C)c1ccccc1C(=O)NCCCCCC(=O)Nc2c(N)cccc2 (Rusche, et al., 2016)	4.447	-0.648	-0.190	b
**276**	N#Cc1ccccc1C(=O)NCCCCCC(=O)Nc2c(N)cccc2	0.426	0.371	0.090	b

	(Rusche, et al., 2016)				
**277**	O=C(C)c1ccccc1C(=O)NCCCCCC(=O)Nc2c(N)cccc2<br>(Rusche, et al., 2016)	2.644	-0.422	-0.370	a
**278**	c1ccccc1C(=O)c(cccc2)c2C(=O)NCCCCCC(=O)Nc3c(N)cccc3<br>(Rusche, et al., 2016)	2.720	-0.435	-0.430	a
**279**	c1ccccc1-c2ccccc2C(=O)NCCCCCC(=O)Nc3c(N)cccc3<br>(Rusche, et al., 2016)	5.732	-0.758	-0.830	a
**280**	FC(F)Oc1ccccc1C(=O)NCCCCCC(=O)Nc2c(N)cccc2<br>(Rusche, et al., 2016)	1.479	-0.170	0.030	b
**281**	COCCOc1ccccc1C(=O)NCCCCCC(=O)Nc2c(N)cccc2<br>(Rusche, et al., 2016)	2.396	-0.379	-0.310	a
**282**	FC(F)(F)c1ccccc1C(=O)NCCCCCC(=O)Nc2c(N)cccc2<br>(Rusche, et al., 2016)	3.564	-0.552	-0.490	a
**283**	Fc1ccccc1C(=O)NCCCCCC(=O)Nc2c(N)cccc2<br>(Rusche, et al., 2016)	1.135	-0.055	-0.230	b
**284**	COc1ccccc1C(=O)NCCCCCC(=O)Nc2c(N)cccc2<br>(Rusche, et al., 2016)	0.674	0.171	-0.080	b
**285**	Brc1ccccc1C(=O)NCCCCCC(=O)Nc2c(N)cccc2<br>(Rusche, et al., 2016)	2.719	-0.434	-0.160	a
**286**	COc1cccc(c12)=NCC=2C(=O)NCCCCCC(=O)Nc3c(N)cccc3<br>(Rusche, et al., 2016)	0.197	0.706	0.500	a
**287**	c1c[nH]c(c12)c(ccc2)C(=O)NCCCCCC(=O)Nc3c(N)cccc3<br>(Rusche, et al., 2016)	0.278	0.556	0.360	b
**288**	c1n[nH]c(c12)cc(cc2)C(=O)NCCCCCC(=O)Nc3c(N)cccc3<br>(Rusche, et al., 2016)	0.582	0.235	0.100	a
**289**	COc(c1)ccc(c12)CC(=N2)C(=O)NCCCCCC(=O)Nc3c(N)cccc3<br>(Rusche, et al., 2016)	0.193	0.714	0.860	a
**290**	c1cc(NC)ccc1C(=O)NCCCCCC(=O)Nc2c(N)cccc2<br>(Rusche, et al., 2016)	0.449	0.348	0.090	b
**291**	C1CC1Nc(cc2)ccc2C(=O)NCCCCCC(=O)Nc3c(N)cccc3<br>(Rusche, et al., 2016)	0.315	0.502	-0.010	b
**292**	C1CN(C)CCN1c(cc2)ccc2C(=O)NCCCCCC(=O)Nc3c(N)cccc3<br>(Rusche, et al., 2016)	0.445	0.352	0.190	b
**293**	c1c(C)ccc(c12)CC(=N2)C(=O)NCCCCCC(=O)Nc3c(N)cccc3<br>(Rusche, et al., 2016)	0.177	0.752	0.650	a
**294**	CCOc(cc1)cc(c12)cc(n2C)C(=O)NCCCCCC(=O)Nc3c(N)cccc3<br>(Rusche, et al., 2016)	0.327	0.485	0.500	a
**295**	Cc1c(C)[nH]c(c12)cc(cc2)C(=O)NCCCCCC(=O)Nc3c(N)cccc3<br>(Rusche, et al., 2016)	0.201	0.697	0.750	a
**296**	c1c(C)ccc(c12)n[nH]c2C(=O)NCCCCCC(=O)Nc3c(N)cccc3<br>(Rusche, et al., 2016)	0.220	0.658	0.720	a

**297**	c1c(Cl)ccc(c12)=NCC=2C(=O)NCCCCCC(=O)Nc3c(N)cccc3<br>(Rusche, et al., 2016)	0.263	0.580	0.490	a
**298**	c1c[nH]c(c12)cc(cc2)C(=O)NCCCCCC(=O)Nc3c(N)cccc3<br>(Rusche, et al., 2016)	0.234	0.631	0.120	b
**299**	c1ccccc1S(=O)(=O)NCCCCCC(=O)Nc2c(N)cccc2<br>(Rusche, et al., 2016)	7.00	-0.845	-0.850	a
**300**	c1cc(C)ccc1C(=O)NCCCCCCC(=O)Nc2c(N)cccc2<br>(Rusche, et al., 2016)	0.19	0.721	0.220	b
**301**	c1cccc(c12)cc(o2)C(=O)NCCCCCC(=O)Nc3c(N)cc(F)cc3<br>(Rusche, et al., 2016)	0.76	0.119	0.30	a
**302**	c1cc(C)ccc1C(=O)NCCCCCC(=O)Nc2c(O)cc(F)cc2<br>(Rusche, et al., 2016)	1.00	0.00	-0.11	b
**303**	n1ccccc1C(=O)NCCCCCC(=O)Nc2c(N)cc(F)cc2<br>(Rusche, et al., 2016)	3.890	-0.59	-0.55	a
**304**	c1ncccc1C(=O)NCCCCCCC(=O)Nc2cc(ccc2N)-c3cccs3<br>(Rusche, et al., 2016)	0.021	1.678	1.72	a*
**305**	c1cc(F)ccc1C(=O)NCCCCCC(=O)Nc2ccccc2N<br>(Rusche, et al., 2016)	1.8	-0.255	-0.03	a
**306**	c1cc(Cl)ccc1C(=O)NCCCCCC(=O)Nc2ccccc2N<br>(Rusche, et al., 2016)	0.7	0.155	0.25	b
**307**	c1cc(OC)ccc1C(=O)NCCCCCC(=O)Nc2ccccc2N<br>(Rusche, et al., 2016)	1.7	-0.230	-0.18	a
**308**	c1c(Cl)cccc1C(=O)NCCCCCC(=O)Nc2ccccc2N<br>(Rusche, et al., 2016)	2	-0.301	-0.11	a
**309**	c1cc(N(C)C)ccc1C(=O)NCCCCCC(=O)Nc2ccccc2N<br>(Rusche, et al., 2016)	1.00	0.00	0.160	b
**310**	c1cc(C(C)(C)C)ccc1C(=O)NCCCCCC(=O)Nc2ccccc2N<br>(Rusche, et al., 2016)	0.6	0.222	0.45	b
**311**	c1cc(C(F)(F)F)ccc1C(=O)NCCCCCC(=O)Nc2ccccc2N<br>(Rusche, et al., 2016)	1.1	-0.041	-0.08	a
**312**	c1cc([N+]([O-])=O)ccc1C(=O)NCCCCCC(=O)Nc2ccccc2N<br>(Rusche, et al., 2016)	1.2	-0.079	0.05	b
**313**	[O-][N+](=O)c(c1)cccc1C(=O)NCCCCCC(=O)Nc2ccccc2N<br>(Rusche, et al., 2016)	0.8	0.097	0.01	b
**314**	FC(F)(F)c(c1)cccc1C(=O)NCCCCCC(=O)Nc2ccccc2N<br>(Rusche, et al., 2016)	0.7	0.155	0.00	a
**315**	c1cc(C#N)ccc1C(=O)NCCCCCC(=O)Nc2ccccc2N<br>(Rusche, et al., 2016)	0.7	0.155	-0.14	b
**316**	c1c(Cl)cc(Cl)cc1C(=O)NCCCCCC(=O)Nc2ccccc2N<br>(Rusche, et al., 2016)	0.4	0.398	0.270	b
**317**	c1ccsc1C(=O)NCCCCCC(=O)Nc2ccccc2N	0.649	0.188	0.13	a

	(Rusche, et al., 2016)				
**318**	c1cc(C)ccc1C(=O)NCCCCCC(=O)Nc2cc(c(F)cc2N)-n3cccn3<br>(Rusche, et al., 2016)	0.442	0.355	0.210	a
**319**	O1COc(c12)c(N)c(cc2)NC(=O)CCCCCNC(=O)c3ccc(C)cc3<br>(Rusche, et al., 2016)	2.89	-0.461	-0.5	a
**320**	n1ccsc1C(=O)NCCCCCC(=O)Nc2ccccc2N<br>(Rusche, et al., 2016)	0.973	0.012	-0.030	a
**321**	n1c(C)csc1C(=O)NCCCCCC(=O)Nc2ccccc2N<br>(Rusche, et al., 2016)	0.721	0.142	0.470	a
**322**	n1cc(C)sc1C(=O)NCCCCCC(=O)Nc2ccccc2N<br>(Rusche, et al., 2016)	0.816	0.088	-0.100	a
**323**	c1cc(Cl)cc(Cl)c1C(=O)NCCCCCC(=O)Nc2ccccc2N<br>(Rusche, et al., 2016)	2.270	-0.356	-0.230	a
**324**	c1cc(S(=O)(=O)C)ccc1S(=O)NCCCCCC(=O)Nc2c(N)cccc2<br>(Rusche, et al., 2016)	0.57	0.244	0.140	b
**325**	c1cc(S(=O)(=O)N)ccc1C(=O)NCCCCCC(=O)Nc2c(N)cccc2<br>(Rusche, et al., 2016)	1.104	-0.043	-0.03	a
**326**	c1cnccc1C(=O)NCCCCCC(=O)Nc2c(N)cccc2<br>(Rusche, et al., 2016)	1.260	-0.100	-0.150	b
**327**	n1ccncc1C(=O)NCCCCCC(=O)Nc2c(N)cccc2<br>(Rusche, et al., 2016)	2.045	-0.311	-0.410	a
**328**	c1nnccc1C(=O)NCCCCCC(=O)Nc2c(N)cccc2<br>(Rusche, et al., 2016)	2.565	-0.409	-0.460	a
**329**	c1ccoc1C(=O)NCCCCCC(=O)Nc2ccccc2N<br>(Rusche, et al., 2016)	0.99	0.004	0.130	a
**330**	c1cocc1C(=O)NCCCCCC(=O)Nc2ccccc2N<br>(Rusche, et al., 2016)	1.24	-0.093	-0.040	a
**331**	c1cscc1C(=O)NCCCCCC(=O)Nc2ccccc2N<br>(Rusche, et al., 2016)	0.76	0.119	0.000	b
**332**	[nH]1cccc1C(=O)NCCCCCC(=O)Nc2c(N)cccc2<br>(Rusche, et al., 2016)	0.463	0.334	0.240	a
**333**	[nH]1cnnc1C(=O)NCCCCCC(=O)Nc2c(N)cccc2<br>(Rusche, et al., 2016)	2.689	-0.43	-0.20	a
**334**	Nc1ccccc1NC(=O)CCCCCNC(=O)c2oncc2<br>(Rusche, et al., 2016)	1.372	-0.137	-0.230	a
**335**	Nc1ccccc1NC(=O)CCCCCNC(=O)c2ncsc2<br>(Rusche, et al., 2016)	1.039	-0.017	-0.090	a
**336**	Nc1ccccc1NC(=O)CCCCCNC(=O)c2cc(ncc2)N3CCCCC3<br>(Rusche, et al., 2016)	0.238	0.623	0.620	b
**337**	Nc1ccccc1NC(=O)CCCCCNC(=O)c2n[nH]c(c2)-c3ccccc3<br>(Rusche, et al., 2016)	0.388	0.411	0.490	b

**338**	Nc1ccccc1NC(=O)CCCCCNC(=O)c(cc2)cc(c23)OCCO3<br>(Rusche, et al., 2016)	0.754	0.123	0.420	b
**339**	Nc1ccccc1NC(=O)CCCCCNC(=O)c(c2)oc(c23)cccc3<br>(Rusche, et al., 2016)	0.418	0.379	0.440	a
**340**	Nc1ccccc1NC(=O)CCCCCNC(=O)c(cc2)cc(c23)scn3<br>(Rusche, et al., 2016)	0.433	0.364	0.570	b
**341**	Nc1ccccc1NC(=O)CCCCCNC(=O)CCC(=O)c2cccs2<br>(Rusche, et al., 2016)	0.78	0.108	0.060	a
**342**	Nc1ccccc1NC(=O)CCCCCNC(=O)c(cc2)cc(c23)non3<br>(Rusche, et al., 2016)	1.238	-0.093	-0.150	a
**343**	Nc1ccccc1NC(=O)CCCCCNC(=O)c(cc2)cc(c23)nccn3<br>(Rusche, et al., 2016)	1.027	-0.012	-0.220	a
**344**	Nc1ccccc1NC(=O)CCCCCNC(=O)c(cc2)cc(c23)nccc3<br>(Rusche, et al., 2016)	0.617	0.210	-0.190	b
**345**	Nc1ccccc1NC(=O)CCCCCNC(=O)c(c2)ccc(c23)cccc3<br>(Rusche, et al., 2016)	0.187	0.728	0.550	a
**346**	Nc1ccccc1NC(=O)CCCCCNC(=O)c(cc2)cc(c23)NC(=O)C3<br>(Rusche, et al., 2016)	0.725	0.140	0.120	a
**347**	Nc1ccccc1NC(=O)CCCCCNC(=O)c2cc(ccc2)-c3nnn[nH]3<br>(Rusche, et al., 2016)	0.466	0.332	0.290	a
**348**	Nc1ccccc1NC(=O)CCCCCNC(=O)c2ccc(cc2)-c3nnn[nH]3<br>(Rusche, et al., 2016)	0.371	0.431	0.440	a
**349**	Nc1ccccc1NC(=O)CCCCCNC(=O)CCc2oc(cn2)-c3ccccc3<br>(Rusche, et al., 2016)	0.312	0.506	0.460	a
**350**	Nc1ccccc1NC(=O)CCCCCNC(=O)c(no2)cc2-c3cscc3<br>(Rusche, et al., 2016)	0.484	0.315	0.370	a
**351**	Nc1ccccc1NC(=O)CCCCCNC(=O)C(=N2)Cc(c23)cc(cc3)OC<br>(Rusche, et al., 2016)	0.258	0.588	0.720	b
**352**	Nc1ccccc1NC(=O)CCCCCNC(=O)c2noc(c2)C3CC3<br>(Rusche, et al., 2016)	0.673	0.172	0.040	b
**353**	Nc1ccccc1NC(=O)CCCCCNC(=O)c2n[nH]c(c23)cccc3<br>(Rusche, et al., 2016)	0.107	0.971	0.310	b
**354**	Nc1ccccc1NC(=O)CCCCCNC(=O)c(nc2)cc(c23)cccc3<br>(Rusche, et al., 2016)	0.264	0.578	0.550	a
**355**	Nc1ccccc1NC(=O)CCCCCNC(=O)c(cn2)cc(c23)cccc3<br>(Rusche, et al., 2016)	0.479	0.320	0.160	b
**356**	Nc1ccccc1NC(=O)CCCCCNC(=O)c(cnn2)c(c23)cccc3<br>(Rusche, et al., 2016)	4.312	-0.635	-0.490	a
**357**	Nc1ccccc1NC(=O)CCCCCNC(=O)c(cn2)nc(c23)cccc3<br>(Rusche, et al., 2016)	0.388	0.411	0.410	a
**358**	Nc1ccccc1NC(=O)CCCCCNC(=O)c2csc(n2)-c3ccncc3	0.3	0.523	0.580	b

	(Rusche, et al., 2016)				
**359**	Nc1ccccc1NC(=O)CCCCCNC(=O)c2sc(nc2C)-c3cnccc3<br>(Rusche, et al., 2016)	0.352	0.453	0.430	b
**360**	Nc1ccccc1NC(=O)CCCCCNC(=O)c2ccc(cc2)-n3cccc3<br>(Rusche, et al., 2016)	0.258	0.588	0.020	a
**361**	Nc1ccccc1NC(=O)CCCCCNC(=O)C2CCN(CC2)c3ccncc3<br>(Rusche, et al., 2016)	1.725	-0.237	-0.290	a
**362**	Nc1ccccc1NC(=O)CCCCCNC(=O)c2c(C)nc(s2)-c3ccccn3<br>(Rusche, et al., 2016)	0.483	0.316	0.450	b
**363**	Nc1ccccc1NC(=O)CCCCNC(=O)C(=N2)Cc(c23)cc(cc3)OC<br>(Rusche, et al., 2016)	3.709	-0.569	-0.630	a
**364**	Nc1ccccc1NC(=O)CCCCNC(=O)c(c2)ccc(c23)ncs3<br>(Rusche, et al., 2016)	11.39	-1.057	-1.150	a
**365**	Nc1ccccc1NC(=O)CCCCNC(=O)c2nc(sc2)-c3ccncc3<br>(Rusche, et al., 2016)	2.766	-0.442	-0.510	a
**366**	Nc1ccccc1NC(=O)CCCCNC(=O)c2ccnc(c2)N3CCCCC3<br>(Rusche, et al., 2016)	18.24	-1.261	-1.220	a
**367**	Nc1ccccc1NC(=O)CCCCNC(=O)c(c2)ccc(c23)OCCO3<br>(Rusche, et al., 2016)	4.615	-0.664	-0.050	b
**368**	Nc1ccccc1NC(=O)CCCCNC(=O)C2=NN=C(C2)c3ccccc3<br>(Rusche, et al., 2016)	2.026	-0.307	0.060	b
**369**	Nc1ccccc1NC(=O)CCCCNC(=O)c2cc(no2)-c(cn3)cn3C<br>(Rusche, et al., 2016)	7.274	-0.862	-0.800	a
**370**	c1cc(C)ccc1NC(=O)CCCCCC(=O)Nc2ccccc2N<br>(Rusche, et al., 2016)	3.050	-0.484	-0.510	a

Table S2. Experimental (Observed column) and predicted (Predicted column) activity pIC_{50} for compounds included in the external test set. (Note: * compounds used for the decoys generation).

	Compounds	Observed IC_{50}(μM)	Observed pIC_{50}	Predicted pIC_{50}	Note
**371**	Nc1ccccc1NC(=O)c2ccc(cc2)CCNC(=O)/C=C/c(cn3)n(c34)cccc4 (Li et al., 2015)	0.217	0.66	0.34	
**372**	Nc1ccccc1NC(=O)c2ccc(cc2)CCNC(=O)/C=C/c(c(n3)C)n(c34)cccc4 (Li et al., 2015)	0.169	0.77	0.35	
**373**	Nc1cc(F)ccc1NC(=O)c2ccc(cc2)CNC(=O)/C=C/c(cn3)n(c34)nccc4 (Li et al., 2015)	0.131	0.81	0.28	
**374**	Nc1ccccc1NC(=O)c2ccc(cc2)CNc3nc(ccn3)-c(cn4)n(c45)ccnc5 (Li et al., 2013)	0.110	0.96	0.81	
**375**	Nc1ccccc1NC(=O)c2ccc(cc2)CNc3nc(ccn3)-c(c(n4)C)n(c45)cccc5 (Li et al., 2013)	0.108	0.97	0.69	
**376**	Nc1ccccc1NC(=O)c2ccc(cc2)CNc3nc(ccn3)-c(c(n4)C)n(c45)cccn5 (Li et al., 2013)	0.127	0.90	0.67	
**377**	Nc1ccccc1NC(=O)c2ccc(cc2)CNc3nc(ccn3)-c(c(n4)C)n(c45)ccnc5 (Li et al., 2013)	0.097	1.01	0.69	
**378**	Nc1ccccc1NC(=O)c2ccc(cc2)CCNc3nc(ccn3)-c(c(n4)C)n(c45)cccn5 (Li et al., 2013)	0.154	0.81	0.96	
**379**	Nc1ccc(F)cc1NC(=O)c2ccc(cc2)CNc3nc(ccn3)-c(c(n4)C)n(c45)cccc5 (Li et al., 2013)	0.097	1.01	0.75	
**380**	Nc1cc(F)ccc1NC(=O)c2ccc(cc2)CNc3nc(ccn3)-c(c(n4)C)n(c45)cccc5 (Li et al., 2013)	0.135	0.87	0.71	
**381**	Nc1ccccc1NC(=O)c2ccc(cc2)CNc3nc(ccn3)-c(c(n4)C)n(c45)cc(Br)cc5 (Li et al., 2013)	0.125	0.90	0.73	
**382**	Nc1ccccc1NC(=O)c2ccc(cc2)CSc3[nH]ccn3 (Frechette et al., 2008)	0.5	0.30	0.10	
**383**	Nc1ccccc1NC(=O)c2ccc(cc2)CSc3n(C)ccn3 (Frechette et al., 2008)	1	0.00	0.11	
**384**	Nc1ccccc1NC(=O)c2ccc(cc2)CNc3sc(Br)cn3 (Frechette et al., 2008)	0.4	0.40	0.31	
**385**	Nc1ccccc1NC(=O)c2ccc(cc2)CNc3n[nH]c(n3)SC (Frechette et al., 2008)	0.2	0.70	0.62	
**386**	Nc1ccccc1NC(=O)c2ccc(cc2)CNc3cc([nH]n3)-c4ccccc4 (Frechette et al., 2008)	0.07	1.15	0.82	
**387**	Nc1ccccc1NC(=O)c2ccc(cc2)CNc3sc(cn3)-c4ccc(Cl)cc4 (Frechette et al., 2008)	0.3	0.52	0.52	
**388**	Nc1ccccc1NC(=O)c2ccc(cc2)CSc(s3)nc(c34)ccc(c4)NCc5cccnc5 (Frechette et al., 2008)	0.8	0.1	0.31	

**389**	Nc1ccccc1NC(=O)c2ccc(cc2)CSC3=N[C@H](CN3)c4ccccc4 (Marson et al., 2015)	0.24	0.62	0.31	
**390**	Nc1ccccc1NC(=O)c2ccc(cc2)CSC3=N[C@H](CN3)c4ccccc4 (Marson et al., 2015)	0.38	0.420	0.067	
**391**	Nc1ccccc1NC(=O)c2ccc(cc2)CSC3=N[C@H](CO3)Cc4ccccc4 (Marson et al., 2015)	0.70	0.15	0.23	
**392**	Nc1ccccc1NC(=O)c2ccc(cc2)CNC3=N[C@H](CO3)c4ccccc4 (Marson et al., 2015)	0.53	0.27	0.42	
**393**	Nc1ccccc1NC(=O)c2ccc(cc2)CNC3=NC(CO3)c4ccc(F)cc4 (Marson et al., 2015)	0.19	0.72	0.33	
**394**	Nc1ccccc1NC(=O)c2ccc(cc2)CNC3=NC(CO3)c4cc(F)ccc4 (Marson et al., 2015)	0.24	0.62	0.35	
**395**	Nc1ccccc1NC(=O)c2ccc(cc2)CNC3=NC(CO3)c4ccc(O)cc4 (Marson et al., 2015)	0.29	0.54	0.34	
**396**	Nc1ccccc1NC(=O)c2ccc(cc2)CNC3=NC(CO3)c4cccnc4 (Marson et al., 2015)	0.26	0.58	0.25	
**397**	Nc1ccccc1NC(=O)c2ccc(cc2)CNC3=N[C@H](CO3)Cc4ccccc4 (Marson et al., 2015)	0.20	0.50	0.57	
**398**	Nc1ccccc1NC(=O)c2ccc(cc2)CNC3=NC[C@@H](O3)c4ccccc4 (Marson et al., 2015)	0.39	0.41	0.36	
**399**	Nc1ccccc1NC(=O)c2ccc(cc2)CNC3=N[C@@H](CO3)Cc4c[nH]cn4 (Marson et al., 2015)	0.13	0.89	0.48	
**400**	Fc1ccc(c(c1)N)NC(=O)c2ccc(cc2)NC(=O)NCc3ccccc3 (Zhu et al., 2017)	0.772	0.11	0.41	
**401**	Nc1ccccc1NC(=O)c2ccc(cc2)CNC(=O)Nc3cc(C(F)(F)F)ccc3 (Zhu et al., 2017)	0.182	0.74	0.37	
**402**	Nc1ccccc1NC(=O)c2ccc(cc2)CNC(=O)Nc3ccc(C(F)(F)F)cc3 (Zhuet et al., 2017)	0.190	0.72	0.29	
**403**	Nc1ccccc1NC(=O)c2ccc(cc2)CNC(=O)Nc3ccc(Cl)cc3 (Zhu et al., 2017)	0.234	0.63	0.35	
**404**	Nc1ccccc1NC(=O)c2ccc(cc2)CNC(=O)Nc3cc(Cl)c(F)cc3 (Zhu et al., 2017)	0.624	0.20	0.31	
**405**	Nc1ccccc1NC(=O)c2ccc(cc2)CNC(=O)Nc3cc(C(F)(F)F)c(Cl)cc3 (Zhu et al., 2017)	0.211	0.67	0.30	
**406**	Nc1ccccc1NC(=O)c2ccc(cc2)CNC(=O)NCc3ccccc3 (Zhu et al., 2017)	0.123	0.91	0.59	
**407**	Fc1ccc(c(c1)N)NC(=O)c2ccc(cc2)CNC(=O)NCc3ccccc3 (Zhu et al., 2017)	1.14	-0.06	0.034	
**408**	c1cccc(N)c1NC(=O)c2ccc(cc2)CNc3cc(c(C)cc3)Nc4nc(ccn4)-c5cccnc5 (Mahboobi et al., 2009)	0.208	0.68	0.83	

**409**	c1ncccc1-c(cn2)cnc2Nc3cc(ccc3)NC(=O)c4cccc(c4)C(=O)Nc5c(N)cccc5 (Mahboobi et al., 2009)	0.76	0.12	0.170	
**410**	c1ncccc1-c(cn2)cnc2Nc3cc(ccc3C)NC(=O)c4cccc(c4)C(=O)Nc5c(N)cccc5 (Mahboobi et al., 2009)	0.27	0.57	0.161	
**411**	c1ncccc1-c2nc(sc2)Nc3cc(ccc3)NC(=O)c4cccc(c4)C(=O)Nc5c(N)cccc5 (Mahboobi et al., 2009)	0.23	0.64	0.77	
**412**	c1ncccc1-c2nc(sc2)Nc3cc(ccc3)NC(=O)c4ccc(s4)C(=O)Nc5c(N)cccc5 (Mahboobi et al., 2009)	0.47	0.33	0.16	
**413**	c1cncc(n12)ncc2-c(n3)ccnc3NCc4ccc(cc4)C(=O)Nc5ccccc5N (Li et al., 2013)	0.110	0.96	0.81	
**414**	c1cccc(n12)nc(C)c2-c(n3)ccnc3NCc4ccc(cc4)C(=O)Nc5ccccc5N (Li et al., 2013)	0.108	0.97	0.75	
**415**	c1ccnc(n12)nc(C)c2-c(n3)ccnc3NCc4ccc(cc4)C(=O)Nc5ccccc5N (Li et al., 2013)	0.127	0.90	0.64	
**416**	c1cncc(n12)nc(C)c2-c(n3)ccnc3NCc4ccc(cc4)C(=O)Nc5ccccc5N (Li et al., 2013)	0.097	1.01	0.69	
**417**	Nc1ccccc1NC(=O)c(cc2)ccc2CCNc3nccc(n3)-c4c(C)nc(n45)cccc5 (Li et al., 2013)	0.131	0.88	1.04	
**418**	Nc1ccccc1NC(=O)c(cc2)ccc2CCNc3nccc(n3)-c4cnc(n45)nccc5 (Li et al., 2013)	0.154	0.81	0.85	
**419**	c1cccc(n12)nc(C)c2-c(n3)ccnc3NCc4ccc(cc4)C(=O)Nc5cc(F)ccc5N (Li et al., 2013)	0.097	1.01	0.77	
**420**	c1cccc(n12)nc(C)c2-c(n3)ccnc3NCc4ccc(cc4)C(=O)Nc5ccc(F)cc5N (Li et al., 2013)	0.135	0.90	0.74	
**421**	c1c(Br)ccc(n12)nc(C)c2-c(n3)ccnc3NCc4ccc(cc4)C(=O)Nc5ccccc5N (Li et al., 2013)	0.125	0.90	0.68	
**422**	c1ccncc1COC(=O)NCc2ccc(cc2)C(=O)Nc3c(N)ccc(c3)-c4ccsc4 (Harrington et al., 2013)	0.0058	2.22	1.75	*
**423**	c1ccncc1COC(=O)NCc2ccc(cc2)C(=O)Nc3c(O)ccc(c3)-c4ccccc4 (Harrington et al., 2013)	0.02	1.70	1.49	*
**424**	CC(C)(C)OC(=O)NCc1ccc(cc1)C(=O)Nc2c(O)ccc(c2)-c3ccccc3 (Harrington et al., 2013)	0.07	1.15	1.50	
**425**	NCc1ccc(cc1)C(=O)Nc2c(O)ccc(c2)-c3ccccc3 (Harrington et al., 2013)	0.028	1.55	1.23	*
**426**	COC(=O)NCc1ccc(cc1)C(=O)Nc2cc(ccc2N)-c3ccccc3 (Harrington et al., 2013)	0.017	1.77	1.34	*
**427**	CCCOC(=O)NCc1ccc(cc1)C(=O)Nc2c(N)ccc(c2)-c3ccccc3 (Harrington et al., 2013)	0.029	1.54	1.36	*

**428**	CC(C)COC(=O)NCc1ccc(cc1)C(=O)Nc2c(N)ccc(c2)-c3ccccc3 (Harrington et al., 2013)	0.045	1.35	1.35	
**429**	c1ccncc1CCC(=O)NCc2ccc(cc2)C(=O)Nc3cc(ccc3N)-c4ccccc4 (Harrington et al., 2013)	0.011	1.96	1.49	*
**430**	c1ccccc1COC(=O)NCc2ccc(cc2)C(=O)Nc3cc(ccc3N)-c4ccccc4 (Harrington et al., 2013)	0.058	1.24	1.51	
**431**	CC(C)OC(=O)NCc1ccc(cc1)C(=O)Nc2c(N)ccc(c2)-c3cccs3 (Harrington et al., 2013)	0.019	1.72	1.30	*
**432**	CCCOC(=O)NCc1ccc(cc1)C(=O)Nc2c(N)ccc(c2)-c3cccs3 (Harrington et al., 2013)	0.019	1.72	1.47	*
**433**	CC(C)COC(=O)NCc1ccc(cc1)C(=O)Nc2c(N)ccc(c2)-c3cccs3 (Harrington et al., 2013)	0.031	1.51	1.23	*
**434**	c1ccccc1OC(=O)NCc2ccc(cc2)C(=O)Nc3c(N)ccc(c3)-c4cccs4 (Harrington et al., 2013)	0.077	1.11	1.15	
**435**	c1ccccc1COC(=O)NCc2ccc(cc2)C(=O)Nc3c(N)ccc(c3)-c4cccs4 (Harrington et al., 2013)	0.053	1.27	1.33	
**436**	CC(=O)NCc1ccc(cc1)C(=O)Nc2c(N)ccc(c2)-c3ccccc3 (Harrington et al., 2013)	0.018	1.74	1.33	*
**437**	CCC(=O)NCc1ccc(cc1)C(=O)Nc2cc(ccc2N)-c3ccccc3 (Harrington et al., 2013)	0.017	1.77	1.33	*
**438**	CC(C)(C)C(=O)NCc1ccc(cc1)C(=O)Nc2c(N)ccc(c2)-c3ccccc3 (Harrington et al., 2013)	0.025	1.60	1.11	*
**439**	C1CCCCC1C(=O)NCc2ccc(cc2)C(=O)Nc3cc(ccc3N)-c4ccccc4 (Harrington et al., 2013)	0.042	1.38	1.24	
**440**	C1CCCC1C(=O)NCc2ccc(cc2)C(=O)Nc3c(N)ccc(c3)-c4ccccc4 (Harrington et al., 2013)	0.021	1.68	1.27	*
**441**	c1ccccc1CC(=O)NCc2ccc(cc2)C(=O)Nc3c(N)ccc(c3)-c4ccccc4 (Harrington et al., 2013)	0.072	1.14	1.21	
**442**	C1CCCCC1C(=O)NCc2ccc(cc2)C(=O)Nc3cc(ccc3N)-c4cccs4 (Harrington et al., 2013)	0.034	1.47	1.12	*
**443**	C1CCCC1C(=O)NCc2ccc(cc2)C(=O)Nc3c(N)ccc(c3)-c4cccs4 (Harrington et al., 2013)	0.020	1.70	1.23	*
**444**	c1cccnc1CC(=O)NCc2ccc(cc2)C(=O)Nc3c(N)ccc(c3)-c4ccccc4 (Harrington et al., 2013)	0.019	1.72	1.65	*
**445**	c1ccncc1CCC(=O)NCc2ccc(cc2)C(=O)Nc3cc(ccc3N)-c4ccccc4 (Harrington et al., 2013)	0.016	1.79	1.50	*
**446**	c1cnccc1CC(=O)NCc2ccc(cc2)C(=O)Nc3c(N)ccc(c3)-c4cccs4 (Harrington et al., 2013)	0.018	1.74	1.24	*
**447**	c1ccncc1CCC(=O)NCc2ccc(cc2)C(=O)Nc3c(N)ccc(c3)-c4cccs4 (Harrington et al., 2013)	0.013	1.89	1.51	*
**448**	C1CCN[C@@H]1C(=O)NCc2ccc(cc2)C(=O)Nc3c(N)ccc(c3)-c4cccs4	0.018	1.74	1.44	*

	(Harrington et al., 2013)				
**449**	C1CCN[C@@H]1C(=O)NCc2ccc(cc2)C(=O)Nc3c(N)ccc(c3)-c4ccccc4<br>(Harrington et al., 2013)	0.024	1.62	1.26	*
**450**	c1cccnc1COC(=O)NCc2ccc(cc2)C(=O)Nc3cc(ccc3N)-c4ccccc4<br>(Harrington et al., 2013)	0.020	1.70	1.27	*
**451**	c1cnccc1NCc2ccc(cc2)C(=O)Nc3cc(ccc3N)-c4ccccc4<br>(Harrington et al., 2013)	0.010	2	1.61	*
**452**	c1cnccc1N(C)Cc2ccc(cc2)C(=O)Nc3cc(ccc3N)-c4ccccc4<br>(Harrington et al., 2013)	0.010	2	2.02	*
**453**	Cc1n(C)ncc1CNCc2ccc(cc2)C(=O)Nc3cc(ccc3N)-c4ccccc4<br>(Harrington et al., 2013)	0.013	1.89	1.97	*
**454**	c1ccccc1-c2ccc(N)c(c2)NC(=O)c3ccc(cc3)CN(C)CCCNC<br>(Harrington et al., 2013)	0.018	1.74	1.81	*
**455**	c1ccccc1-c2ccc(N)c(c2)NC(=O)c3ccc(cc3)CNCC(C)C<br>(Harrington et al., 2013)	0.035	1.45	1.30	*
**456**	c1ccccc1-c2ccc(N)c(c2)NC(=O)c3ccc(cc3)CNC(C)COC<br>(Harrington et al., 2013)	0.023	1.64	1.40	*
**457**	c1cnccc1NCc2ccc(cc2)C(=O)Nc3cc(ccc3N)-c4ccccc4F<br>(Harrington et al., 2013)	0.023	1.64	1.63	*
**458**	c1cnccc1NCc2ccc(cc2)C(=O)Nc3cc(ccc3N)-c4cccc(F)c4<br>(Harrington et al., 2013)	0.027	1.57	1.67	*
**459**	c1cnccc1NCc2ccc(cc2)C(=O)Nc3cc(ccc3N)-c4cc(cs4)C<br>(Harrington et al., 2013)	0.015	1.82	1.46	*
**460**	c1ccccc1-c2ccc(O)c(c2)NC(=O)c3ccc(cc3)CNC4CC4<br>(Harrington et al., 2013)	0.076	1.12	1.22	
**461**	c1ccccc1NCc2ccc(cc2)C(=O)Nc3cc(ccc3O)-c4ccccc4<br>(Harrington et al., 2013)	0.089	1.05	1.2	
**462**	c1ccccc1-c2ccc(O)c(c2)NC(=O)c3ccc(cc3)CNCC4CC4<br>(Harrington et al., 2013)	0.072	1.14	1.33	
**463**	c1ccncc1CNCc2ccc(cc2)C(=O)Nc3cc(ccc3O)-c4ccccc4<br>(Harrington et al., 2013)	0.032	1.49	1.34	*
**464**	c1cnccc1NCc2ccc(cc2)C(=O)Nc3cc(ccc3O)-c4ccccc4<br>(Harrington et al., 2013)	0.0099	2.0	1.53	*
**465**	c1ccccc1-c2ccc(O)c(c2)NC(=O)c3ccc(cc3)CNCCNC(C)C<br>(Harrington et al., 2013)	0.044	1.36	1.72	
**466**	c1ccccc1-c(ccc2O)cc2NC(=O)c3ccc(cc3)CNCCC(C)C<br>(Harrington et al., 2013)	0.100	1.00	1.33	
**467**	c1ccccc1-c2cc(c(O)cc2)NC(=O)c3ccc(cc3)CN(C)CC<br>(Harrington et al., 2013)	0.031	1.51	1.84	*
**468**	c1ccccc1-c2cc(c(O)cc2)NC(=O)c3ccc(cc3)CN(C)CCCNC<br>(Harrington et al., 2013)	0.018	1.74	1.65	*

**469**	c1cnccc1N(C)Cc2ccc(cc2)C(=O)Nc3c(O)ccc(c3)-c4ccccc4 (Harrington et al., 2013)	0.013	1.89	2.26	*
**470**	c1cnccc1CCN(C)Cc2ccc(cc2)C(=O)Nc3c(O)ccc(c3)-c4ccccc4 (Harrington et al., 2013)	0.033	1.48	1.76	*
**471**	c1ccccc1-c2ccc(O)c(c2)NC(=O)c3ccc(cc3)CNCCN (Harrington et al., 2013)	0.072	1.14	1.43	
**472**	c1ccccc1-c2ccc(O)c(c2)NC(=O)c3ccc(cc3)CNCCCc4ccncc4 (Harrington et al., 2013)	0.100	1.00	1.40	
**473**	c1ccccc1-c2ccc(O)c(c2)NC(=O)c3ccc(cc3)CNCC4CCN(C4)C (Harrington et al., 2013)	0.180	0.74	0.73	
**474**	c1ccccc1-c2ccc(O)c(c2)NC(=O)c3ccc(cc3)CNCCn4nccc4 (Harrington et al., 2013)	0.033	1.48	1.95	*
**475**	c1ccccc1-c2cc(c(O)cc2)NC(=O)c3ccc(cc3)CN(C)Cc4cn(C)nc4 (Harrington et al., 2013)	0.024	1.62	1.88	*
**476**	c1ccccc1[C@@H](COC2=O)N2Cc3ccc(cc3)C(=O)Nc4c(N)ccc(c4)-c5ccccc5 (Harrington et al., 2013)	0.064	1.19	1.27	
**477**	c1ccccc1C[C@H](COC2=O)N2Cc3ccc(cc3)C(=O)Nc4c(N)ccc(c4)-c5ccccc5 (Harrington et al., 2013)	0.089	1.05	1.27	
**478**	c1ccccc1C[C@@H](COC2=O)N2Cc3ccc(cc3)C(=O)Nc4c(N)ccc(c4)-c5ccccc5 (Harrington et al., 2013)	0.096	1.02	1.31	
**479**	c1ccccc1[C@H](COC2=O)N2Cc3ccc(cc3)C(=O)Nc4c(N)ccc(c4)-c5ccccc5 (Harrington et al., 2013)	0.058	1.24	1.36	
**480**	Cn1nnc(c1)CN(C(=O)C)Cc(cc2)ccc2C(=O)Nc3c(N)ccc(c3)-c4sccc4 (Harrington et al., 2013)	0.0095	2.04	1.80	*
**481**	OC(=O)CNCc1ccc(cc1)C(=O)Nc2c(N)ccc(c2)-c3cccs3 (Harrington et al., 2013)	0.034	1.47	1.46	*
**482**	OC(=O)CNCc1ccc(cc1)C(=O)Nc2c(N)ccc(c2)-c3ccsc3 (Harrington et al., 2013)	0.042	1.38	1.63	
**483**	c1ccncc1CN(C(=O)[O-])Cc2ccc(cc2)C(=O)Nc(c3N)cc(cc3)-c4ccccc4 (Harrington et al., 2013)	0.016	1.80	1.51	*

Table S3. Active compounds used for generating a decoys set.

Compound	
**1**	C1CCN1Cc(cn2)cc(C#N)c2-c3ccc(cc3)C(=O)Nc4c(N)cccc4
**2**	c1cccc(N)c1NC(=O)c(cc2)ccc2-c3c(C#N)cc(cn3)CN4CCN(CC4)CC
**3**	c1cccc(N)c1NC(=O)c(cc2)ccc2-c3c(C#N)cc(cn3)CN4CCN(CC4)C(C)C
**4**	c1ccsc1-c(ccc2N)cc2NC(=O)c3ccc(cc3)NC(=O)C
**5**	c1cscc1-c(ccc2N)cc2NC(=O)c3ccc(cc3)NC(=O)C
**6**	c1ccccc1-c(c2)ccc(O)c2NC(=O)c3ccc(cc3)CNc4ccncc4
**7**	c1ccccc1-c(c2)ccc(N)c2NC(=O)c3ccc(cc3)CNc4ccncc4
**8**	c1ccccc1-c(c2)ccc(N)c2NC(=O)c3ccc(cc3)CN(C)c4ccncc4
**9**	c1ccccc1-c(c2)ccc(N)c2NC(=O)c3ccc(cc3)CN(CC4)CCC45NCCC5
**10**	s1cccc1-c(c2)ccc(N)c2NC(=O)c3ccc(cc3)CN(CC4)CCC45NCCC5
**11**	c1sccc1-c(c2)ccc(N)c2NC(=O)c3ccc(cc3)CN(CC4)CCC45NCCC5
**12**	c1ccccc1-c(c2)ccc(N)c2NC(=O)c3ccc(cc3)C(=O)N(CC4)CCC45NCCC5
**13**	C1CNCC12CCN(CC2)c(nc3)ccc3C(=O)Nc4cc(ccc4N)-c5cccs5
**14**	C1CNCC12CCN(CC2)c(nc3)ccc3C(=O)Nc4cc(ccc4N)-c5ccsc5
**15**	C1CN(C(=O)C)CC12CCN(CC2)c(nc3)ccc3C(=O)Nc4cc(ccc4N)-c5cccs5
**16**	C1CN(S(=O)(=O)C)CC12CCN(CC2)c(nc3)ccc3C(=O)Nc4cc(ccc4N)-c5cccs5
**17**	OCCN(CC1)CC12CCN(CC2)c(nc3)ccc3C(=O)Nc4cc(ccc4N)-c5cccs5
**18**	CC(=O)N(C1)CCC12CCCN(C2)c(nc3)ccc3C(=O)Nc4cc(ccc4N)-c5cccs5
**19**	C1NC(=O)NC12CCN(CC2)c(nc3)ccc3C(=O)Nc4cc(ccc4N)-c5cccs5
**20**	C1OC(=O)NC12CCN(CC2)c(nc3)ccc3C(=O)Nc4cc(ccc4N)-c5cccs5
**24**	Nc1ccccc1NC(=O)c2ccc(cc2)C(C(=O)Nc3cccc(c3)C#N)C(=O)Nc4cc(C#N)ccc4
**27**	Nc1ccccc1NC(=O)c2ccc(cc2)C(C(=O)Nc3cc(OC)ccc3)C(=O)Nc4cc(OC)ccc4
**29**	Nc1ccccc1NC(=O)c2ccc(cc2)C(C(=O)Nc(cc3)cc(c34)OCCO4)C(=O)Nc(c5)ccc(c56)OCCO6
**88**	Nc1ccccc1NC(=O)c2ccc(cc2)-c3ncc(s3)CN4CCCCC4
**90**	Nc1ccccc1NC(=O)c2ccc(cc2)-c3ncc(s3)CNCCC
**94**	Nc1ccccc1NC(=O)c2ccc(cc2)C3CCN(CC3)Cc4ccc(cc4)C(=O)NCC
**95**	Nc1ccccc1NC(=O)c2ccc(cc2)C3CCN(CC3)Cc4ccc(cc4)C(=O)NC
**96**	Nc1ccccc1NC(=O)c2ccc(cc2)-c3c(C#N)cc(cn3)N
**97**	C1CCN1Cc(cn2)cc(C)c2-c3ccc(cc3)C(=O)Nc4c(N)cccc4
**98**	C1CCN1Cc(cn2)cc(Cl)c2-c3ccc(cc3)C(=O)Nc4c(N)cccc4
**100**	c1cccc(N)c1NC(=O)c(cc2)ccc2-c3c(C)cc(cn3)CN4CCN(CC4)CC
**101**	c1cccc(N)c1NC(=O)c(cc2)ccc2-c3c(Cl)cc(cn3)CN4CCN(CC4)CC
**103**	c1cccc(N)c1NC(=O)c(cc2)ccc2-c3c(C)cc(cn3)CN4CCN(CC4)C(C)C
**104**	c1cccc(N)c1NC(=O)c(cc2)ccc2-c3c(Cl)cc(cn3)CN4CCN(CC4)C(C)C
**105**	c1cccc(N)c1NC(=O)c(cc2)ccc2-c3c(F)cc(cn3)CN4CCN(CC4)C(C)C
**138**	c1ccccc1-c(ccc2O)cc2NC(=O)c3ccc(cc3)NC(=O)C
**139**	c1ccccc1-c(ccc2N)cc2NC(=O)c3ccc(cc3)NC(=O)C
**150**	c1ccccc1-c(c2)ccc(O)c2NC(=O)c3ccc(cc3)CNCc4cnccc4

**151**	c1ccccc1-c(c2)ccc(O)c2NC(=O)c3ccc(cc3)CNC(C)c4ccncc4
**157**	c1ccccc1-c(c2)ccc(N)c2NC(=O)c3ccc(cc3)CNCc4c(C)n(C)nc4
**158**	c1ccccc1-c(c2)ccc(N)c2NC(=O)c3ccc(cc3)CN(CC4)CCC45CNCC5
**159**	c1cccc(F)c1-c(c2)ccc(N)c2NC(=O)c3ccc(cc3)CN(CC4)CCC45NCCC5
**161**	c1cc(F)ccc1-c(c2)ccc(N)c2NC(=O)c3ccc(cc3)CN(CC4)CCC45NCCC5
**162**	s1cccc1-c(c2)ccc(N)c2NC(=O)c3ccc(cc3)C(=O)N(CC4)CCC45NCCC5
**163**	c1cccc(F)c1-c(c2)ccc(N)c2NC(=O)c3ccc(cc3)C(=O)N(CC4)CCC45NCCC5
**164**	c1cc(F)ccc1-c(c2)ccc(N)c2NC(=O)c3ccc(cc3)C(=O)N(CC4)CCC45NCCC5
**177**	C1CNCC12CCN(CC2)c(nc3)ccc3C(=O)Nc4cc(ccc4N)-c5ccccc5
**179**	C1CNCC12CCN(CC2)c(nc3)ccc3C(=O)Nc4cc(ccc4N)-c5cc[nH]n5
**180**	C1CNCC12CCN(CC2)c(nc3)ccc3C(=O)Nc4cc(ccc4N)-c5cn(cn5)C
**181**	CCNC(=O)N(CC1)CC12CCN(CC2)c(nc3)ccc3C(=O)Nc4cc(ccc4N)-c5cccs5
**182**	n1cccnc1N(CC2)CC23CCN(CC3)c(nc4)ccc4C(=O)Nc5cc(ccc5N)-c6cccs6
**183**	FC(F)(F)CN(CC1)CC12CCN(CC2)c(nc3)ccc3C(=O)Nc4cc(ccc4N)-c5cccs5
**184**	OC(=O)CN(CC1)CC12CCN(CC2)c(nc3)ccc3C(=O)Nc4cc(ccc4N)-c5cccs5
**185**	NC(=O)CN(CC1)CC12CCN(CC2)c(nc3)ccc3C(=O)Nc4cc(ccc4N)-c5cccs5
**186**	CC(=O)N(C1)CCC12CN(CC2)c(nc3)ccc3C(=O)Nc4cc(ccc4N)-c5cccs5
**187**	CC(=O)N(C1)CC12CCN(CC2)c(nc3)ccc3C(=O)Nc4cc(ccc4N)-c5cccs5
**188**	C1CCN(C(=O)C)C12CCN(CC2)c(nc3)ccc3C(=O)Nc4cc(ccc4N)-c5cccs5
**189**	C1CCC(=O)C12CCN(CC2)c(nc3)ccc3C(=O)Nc4cc(ccc4N)-c5cccs5
**190**	C1CCC(O)C12CCN(CC2)c(nc3)ccc3C(=O)Nc4cc(ccc4N)-c5cccs5
**191**	C1CNC(=O)C12CCN(CC2)c(nc3)ccc3C(=O)Nc4cc(ccc4N)-c5cccs5
**192**	C1NC(=O)C12CCN(CC2)c(nc3)ccc3C(=O)Nc4cc(ccc4N)-c5cccs5
**193**	C1C(=O)NCC12CCN(CC2)c(nc3)ccc3C(=O)Nc4cc(ccc4N)-c5cccs5
**194**	N1CNC(=O)C12CCN(CC2)c(nc3)ccc3C(=O)Nc4cc(ccc4N)-c5cccs5
**304**	c1ncccc1C(=O)NCCCCCCC(=O)Nc2cc(ccc2N)-c3cccs3
**422**	c1ccncc1COC(=O)NCc2ccc(cc2)C(=O)Nc3c(N)ccc(c3)-c4ccsc4
**423**	c1ccncc1COC(=O)NCc2ccc(cc2)C(=O)Nc3c(O)ccc(c3)-c4ccccc4
**425**	NCc1ccc(cc1)C(=O)Nc2c(O)ccc(c2)-c3ccccc3
**426**	COC(=O)NCc1ccc(cc1)C(=O)Nc2cc(ccc2N)-c3ccccc3
**427**	CCCOC(=O)NCc1ccc(cc1)C(=O)Nc2c(N)ccc(c2)-c3ccccc3
**429**	c1ccncc1CCC(=O)NCc2ccc(cc2)C(=O)Nc3cc(ccc3N)-c4ccccc4
**431**	CC(C)OC(=O)NCc1ccc(cc1)C(=O)Nc2c(N)ccc(c2)-c3cccs3
**432**	CCCOC(=O)NCc1ccc(cc1)C(=O)Nc2c(N)ccc(c2)-c3cccs3
**433**	c1ccccc1OC(=O)NCc2ccc(cc2)C(=O)Nc3c(N)ccc(c3)-c4cccs4
**436**	CC(=O)NCc1ccc(cc1)C(=O)Nc2c(N)ccc(c2)-c3ccccc3
**437**	CCC(=O)NCc1ccc(cc1)C(=O)Nc2cc(ccc2N)-c3ccccc3
**438**	CC(C)(C)C(=O)NCc1ccc(cc1)C(=O)Nc2c(N)ccc(c2)-c3ccccc3
**440**	C1CCCC1C(=O)NCc2ccc(cc2)C(=O)Nc3c(N)ccc(c3)-c4ccccc4
**442**	C1CCCCC1C(=O)NCc2ccc(cc2)C(=O)Nc3cc(ccc3N)-c4cccs4

**443**	C1CCCC1C(=O)NCc2ccc(cc2)C(=O)Nc3c(N)ccc(c3)-c4cccs4
**444**	c1cccnc1CC(=O)NCc2ccc(cc2)C(=O)Nc3c(N)ccc(c3)-c4ccccc4
**445**	c1ccncc1CCC(=O)NCc2ccc(cc2)C(=O)Nc3cc(ccc3N)-c4ccccc4
**446**	c1cnccc1CC(=O)NCc2ccc(cc2)C(=O)Nc3c(N)ccc(c3)-c4cccs4
**447**	c1ccncc1CCC(=O)NCc2ccc(cc2)C(=O)Nc3c(N)ccc(c3)-c4cccs4
**448**	C1CCN[C@@H]1C(=O)NCc2ccc(cc2)C(=O)Nc3c(N)ccc(c3)-c4cccs4
**449**	C1CCN[C@@H]1C(=O)NCc2ccc(cc2)C(=O)Nc3c(N)ccc(c3)-c4ccccc4
**450**	c1cccnc1COC(=O)NCc2ccc(cc2)C(=O)Nc3cc(ccc3N)-c4ccccc4
**451**	c1cnccc1NCc2ccc(cc2)C(=O)Nc3cc(ccc3N)-c4ccccc4
**452**	c1cnccc1N(C)Cc2ccc(cc2)C(=O)Nc3cc(ccc3N)-c4ccccc4
**453**	Cc1n(C)ncc1CNCc2ccc(cc2)C(=O)Nc3cc(ccc3N)-c4ccccc4
**454**	c1ccccc1-c2ccc(N)c(c2)NC(=O)c3ccc(cc3)CN(C)CCCNC
**455**	c1ccccc1-c2ccc(N)c(c2)NC(=O)c3ccc(cc3)CNCC(C)C
**456**	c1ccccc1-c2ccc(N)c(c2)NC(=O)c3ccc(cc3)CNC(C)COC
**457**	c1cnccc1NCc2ccc(cc2)C(=O)Nc3cc(ccc3N)-c4ccccc4F
**458**	c1cnccc1NCc2ccc(cc2)C(=O)Nc3cc(ccc3N)-c4cccc(F)c4
**459**	c1cnccc1NCc2ccc(cc2)C(=O)Nc3cc(ccc3N)-c4cc(cs4)C
**463**	c1ccncc1CNCc2ccc(cc2)C(=O)Nc3cc(ccc3O)-c4ccccc4
**464**	c1cnccc1NCc2ccc(cc2)C(=O)Nc3cc(ccc3O)-c4ccccc4
**467**	c1ccccc1-c2cc(c(O)cc2)NC(=O)c3ccc(cc3)CN(C)CC
**468**	c1ccccc1-c2cc(c(O)cc2)NC(=O)c3ccc(cc3)CN(C)CCCNC
**469**	c1cnccc1N(C)Cc2ccc(cc2)C(=O)Nc3c(O)ccc(c3)-c4ccccc4
**470**	c1cnccc1CCN(C)Cc2ccc(cc2)C(=O)Nc3c(O)ccc(c3)-c4ccccc4
**474**	c1ccccc1-c2ccc(O)c(c2)NC(=O)c3ccc(cc3)CNCCn4nccc4
**475**	c1ccccc1-c2cc(c(O)cc2)NC(=O)c3ccc(cc3)CN(C)Cc4cn(C)nc4
**480**	Cn1nnc(c1)CN(C(=O)C)Cc(cc2)ccc2C(=O)Nc3c(N)ccc(c3)-c4sccc4
**481**	OC(=O)CNCc1ccc(cc1)C(=O)Nc2c(N)ccc(c2)-c3cccs3
**483**	c1ccncc1CN(C(=O)[O-])Cc2ccc(cc2)C(=O)Nc(c3N)cc(cc3)-c4ccccc4

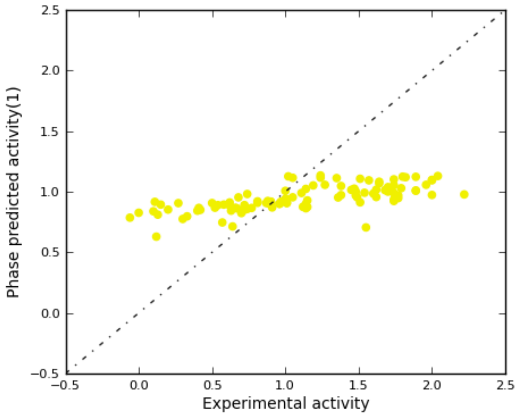

Figure S1. Scatter plot for the predicted (phase predicted activity) and observed (experimental activity) pIC_{50} values (μM) as calculated by the 3D-QSAR model with 1 factor applied to the external test set ($r^2_{ext\_ts}$ = 0.421).

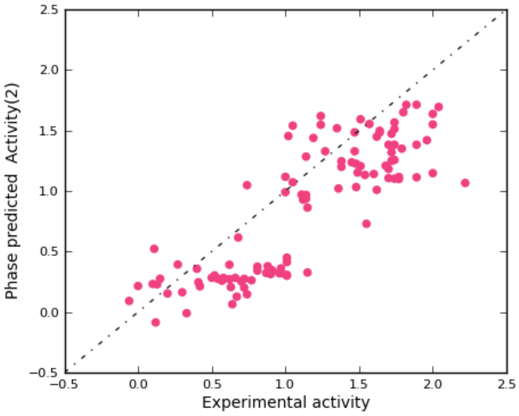

Figure S2. Scatter plot for the predicted (phase predicted activity) and observed (experimental activity) pIC_{50} values (μM) as calculated by the 3D-QSAR model with 2 factors applied to the external test set ($r^2_{ext\_ts}$ = 0.698).

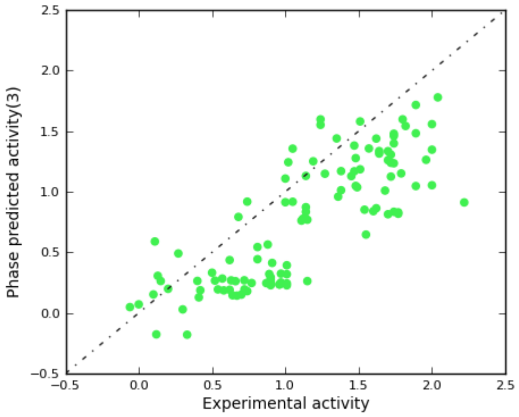

Figure S3. Scatter plot for the predicted (phase predicted activity) and observed (experimental activity) pIC_{50} values (μM) as calculated by the 3D-QSAR model with 3 factors applied to the external test set ($r^2_{ext\_ts}$ = 0.657).

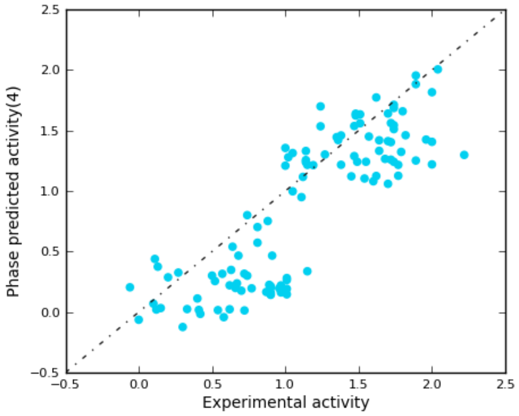

Figure S4. Scatter plot for the predicted (phase predicted activity) and observed (experimental activity) pIC_{50} values (μM) as calculated by the 3D-QSAR model with 4 factors applied to the external test set ($r^2_{ext\_ts}$ = 0.712).

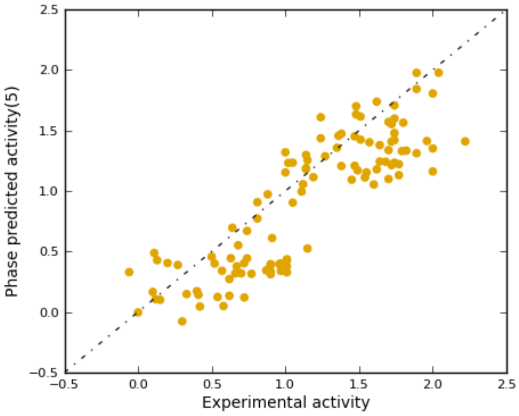

Figure S5. Scatter plot for the predicted (phase predicted activity) and observed (experimental activity) pIC_{50} values (μM) as calculated by the 3D-QSAR model with 5 factors applied to the external test set ($r^2_{ext\_ts}$ = 0.735).

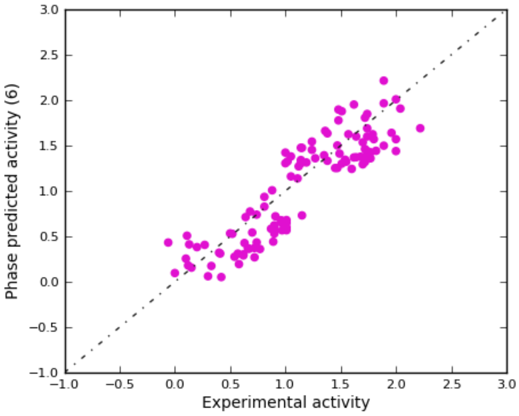

Figure S6. Scatter plot for the predicted (phase predicted activity) and observed (experimental activity) pIC_{50} values (μM) as calculated by the 3D-QSAR model with 6 factors applied to the external test set ($r^2_{ext\_ts}$ = 0.787).

References

Abdizadeh T, Kalani MR, Abnous K, Tayarani-Najaran Z, Khashyarmanesh BZ, Abdizadeh R, Ghodsi R, Hadizadeh F. Design, synthesis and biological evaluation of novel coumarin-based benzamides as potent histone deacetylase inhibitors and anticancer agents, *Eur J Med Chem*, 132 (2017) 42-62.

Andrews DM, Gibson KM, Graham MA, Matusiak ZS, Roberts CA, Stokes ES, Brady MC, Chresta CM. Design and campaign synthesis of pyridine-based histone deacetylase inhibitors, *Bioorg Med Chem Lett*, 18(8) (2008) 2525-2529.

Andrews DM, Stokes ES, Carr GR, Matusiak ZS, Roberts CA, Waring MJ, Brady MC, Chresta CM, East SJ. Design and campaign synthesis of piperidine-and thiazole-based histone deacetylase inhibitors, *Bioorg Med Chem Lett*, 18(8) (2008) 2580-2584.

Fréchette S, Leit S, Woo SH, Lapointe G, Jeannotte G, Moradei O, Paquin I, Bouchain G, Raeppel S, Gaudette F, Zhou N. 4-(Heteroarylaminomethyl)-N-(2-aminophenyl)-benzamides and their analogs as a novel class of histone deacetylase inhibitors, *Bioorg Med Chem Lett*, 18(4) (2008) 1502-1506.

Hamblett CL, Methot JL, Mampreian DM, Sloman DL, Stanton MG, Kral AM, Fleming JC, Cruz JC, Chenard M, Ozerova N, Hitz AM. The discovery of 6-amino nicotinamides as potent and selective histone deacetylase inhibitors, *Bioorg Med Chem Lett*, 17(19) (2007) 5300-5309.

Harrington P, Kattar S, Miller TA, Stanton MG, Tempest P, Witter DJ, inventors; Merck Sharp, Dohme Corp, assignee. 4-carboxybenzylamino derivatives as histone deacetylase inhibitors, *United States patent US*, (2013) 8,389,553; Mar 5.

Hirata Y, Hirata M, Kawaratani Y, Shibano M, Taniguchi M, Yasuda M, Ohmomo Y, Nagaoka Y, Baba K, Uesato S. Anti-tumor activity of new orally bioavailable 2-amino-5-(thiophen-2-yl) benzamide-series histone deacetylase inhibitors, possessing an aqueous soluble functional group as a surface recognition domain, *Bioorg Med Chem Lett*, 22(5) (2012) 1926-1930.

Kattar SD, Surdi LM, Zabierek A, Methot JL, Middleton RE, Hughes B, Szewczak AA, Dahlberg WK, Kral AM, Ozerova N, Fleming JC. Parallel medicinal chemistry approaches to selective HDAC1/HDAC2 inhibitor (SHI-1: 2) optimization, *Bioorg Med Chem Lett*, 19(4) (2009) 1168-1172.

Kiyokawa S, Hirata Y, Nagaoka Y, Shibano M, Taniguchi M, Yasuda M, Baba K, Uesato S. New orally bioavailable 2-aminobenzamide-type histone deacetylase inhibitor possessing a (2-hydroxyethyl)(4-(thiophen-2-yl) benzyl) amino group, *Bioorg Med Chem*, 18(11), (2010) 3925-3933.

Li Y, Wang Y, Xie N, Xu M, Qian P, Zhao Y, Li S. Design, synthesis and antiproliferative activities of novel benzamides derivatives as HDAC inhibitors, *Eur J Med Chem*, 100, (2015) 270-276.

Li Y, Zhou Y, Qian P, Wang Y, Jiang F, Yao Z, Hu W, Zhao Y, Li S. Design, synthesis and bioevalution of novel benzamides derivatives as HDAC inhibitors, *Bioorg Med Chem Lett*, 23(1), (2013).

Mahboobi S, Dove S, Sellmer A, Winkler M, Eichhorn E, Pongratz H, Ciossek T, Baer T, Maier T, Beckers T. Design of chimeric histone deacetylase-and tyrosine kinase-inhibitors: a series of Imatinib hybrides as potent Inhibitors of wild-type and mutant BCR-ABL, PDGF-Rβ, and histone deacetylases, *J Med Chem*, 52(8) (2009) 2265-2279.

Marson CM, Matthews CJ, Atkinson SJ, Lamadema N, Thomas NS. Potent and selective inhibitors of histone deacetylase-3 containing chiral oxazoline capping groups and a N-(2-aminophenyl)-benzamide binding unit, *J Med Chem*, 58(17) (2015) 6803-6818.

Methot JL, Chakravarty PK, Chenard M, Close J, Cruz JC, Dahlberg WK, Fleming J, Hamblett CL, Hamill JE, Harrington P, Harsch A. Exploration of the internal cavity of histone deacetylase (HDAC) with selective HDAC1/HDAC2 inhibitors (SHI-1: 2), *Bioorg Med Chem Lett*, 18(3) (2008) 973-978.

Methot JL, Hamblett CL, Mampreian DM, Jung J, Harsch A, Szewczak AA, Dahlberg WK, Middleton RE, Hughes B, Fleming JC, Wang H. SAR profiles of spirocyclic nicotinamide derived selective HDAC1/HDAC2 inhibitors (SHI-1: 2), *Bioorg Med Chem Lett*, 18(23) (2008) 6104-6109.

Rajak H, Kumar P, Parmar P, Thakur BS, Veerasamy R, Sharma PC, Sharma AK, Gupta AK, Dangi JS. Appraisal of GABA and PABA as linker: design and synthesis of novel benzamide based histone deacetylase inhibitors, *Eur J Med Chem*, 53 (2012) 390-397.

Rusche JR, Peet NP, Hopper AT, inventors; BioMarin Pharmaceutical Inc, assignee. Compositions including 6-aminohexanoic acid derivatives as HDAC inhibitors, *United States patent US*, (2016) 9,265,734; Feb 23.

Siliphaivanh P, Harrington P, Witter DJ, Otte K, Tempest P, Kattar S, Kral AM, Fleming JC, Deshmukh SV,

Valente S, Trisciuoglio D, De Luca T, Nebbioso A, Labella D, Lenoci A, Bigogno C, Dondio G, Miceli M, Brosch G, Del Bufalo D. 1, 3, 4-Oxadiazole-containing histone deacetylase inhibitors: anticancer activities in cancer cells, *J Med Chem*, 57(14) (2014) 6259-6265.

Zhu Y, Chen X, Ran T, Niu J, Zhao S, Lu T, Tang W. Design, synthesis and biological evaluation of urea-based benzamides derivatives as HDAC inhibitors, *Med Chem Res*, 26(11) (2017) 2879-2888.
